# Development of a humanized mouse model of graft-versus-host disease to assess human regulatory T cell function

**DOI:** 10.3389/fimmu.2025.1717133

**Published:** 2025-12-11

**Authors:** Charline Beguin, Oswin Kwan, Grégory Ehx, Stéphanie Humblet-Baron, Murat Cem Köse, Sophie Dubois, Lorenzo Canti, Justine Courtois, Coline Daulne, Jo Caers, Yves Beguin, Caroline Ritacco, Frédéric Baron

**Affiliations:** 1 Hematology Research Unit, Groupe Interdisciplinaire de Génoprotéomique Appliquée (GIGA)-I³, University of Liège, Liège, Belgium; 2 Department of Medicine, Division of Hematology, Centre Hospitalier Universitaire (CHU) of Liège, Liège, Belgium; 3 Walloon Excellence in Life Sciences and Biotechnology (WELBIO) Department, Walloon ExceLlence (WEL) Research Institute, Wavre, Belgium; 4 Department of Microbiology, Immunology and Transplantation, Laboratory of Adaptive Immunology, Katholieke Universiteit (KU) Leuven, Leuven, Belgium

**Keywords:** GVHD, xenogeneic, NSG-HLA-A2/HHD, regulatory T cells, TNF-α, single-cell RNA seq, spectral flow cytometry

## Abstract

**Introduction:**

Regulatory T cells (Treg)-based therapies are increasingly used for treating autoimmune or graft-versus-host (GVHD) disease. Given their low frequency, several approaches aimed at amplifying them or at increasing their immunosuppressive activities are currently investigated. Unfortunately, the impact of these strategies on human Treg function has remained difficult to assess *in vivo*. Here, we report the development of a novel humanized mouse model of allogeneic and xenogeneic GVHD intended to characterize the immunosuppressive activity of human Treg *in vivo*.

**Methods:**

In this model, GVHD is induced by injecting CD25-depleted HLA-A2^−^ peripheral blood mononuclear cells (PBMC) into irradiated NSG-HLA-A2-HHD mice. The CD25^+^ (Treg) fraction is maintained *in vitro* for 48h during which Treg-promoting treatments can be tested before infusion into mice. We took advantage of this model to investigate whether tumor necrosis factor alpha (TNF-α) priming of Treg would increase their suppressive function and improve their ability at preventing xenogeneic GVHD, after assessing the effect of TNF-α on Treg *in vitro*.

**Results:**

*In vitro*, single cell RNAseq analyses showed that eleven Hallmark pathways, including Interferon response, IL-6-JAK-STAT3 signaling, mTORC1 signaling, and TNF-α signaling via NF-κB, were significantly upregulated in Treg following TNF-α priming. *In vivo*, Treg infusion resulted in higher Treg levels, lower counts of human cells, lower conventional CD4^+^ (Tconv) and CD8^+^ T-cell counts, lower KI67 and HLA-DR expression by Tconv and lower Granzyme B expression by CD8^+^ T-cells in peripheral blood. No significant impact of TNF-α priming of Treg on survival was observed.

**Discussion:**

These results emphasize the importance of reliable techniques to assess Treg *in vivo* as efficient methods to activate them *in vitro* do not always result in an enhanced function in the *in vivo* setting. In summary, we present here the development of a novel humanized model of GVHD designed to evaluate the *in vivo* functionality of human Treg. Taking advantage of that model, we observed that TNF-α priming of human Treg did not increase their suppressive activity *in vivo*.

## 1 Introduction

Regulatory T cells (Treg, a subpopulation of CD4^+^ T-cells constitutively expressing CD25 and FoxP3) play an important role in immune homeostasis, preventing autoimmunity (1). Indeed, their absence leads to the lethal immune deficiency, polyendocrinopathy, enteropathy, X-linked (IPEX) syndrome (2). In contrast, several preclinical studies have provided proof of concept that Treg infusion prevented or even reverted autoimmunity in diabetes or collagen-induced arthritis (3). In addition, in the setting of allogeneic hematopoietic stem cell transplantation (allo-HCT), Treg depletion of the graft (via CD25 depletion) has been shown to exacerbate graft-versus-host disease (GVHD), a life-threatening complication of allo-HCT during which donor immune cells attack host healthy tissues (4, 5) (6, 7). On the opposite, co-transplantation of Treg with the graft at conventional T-cell (Tconv)/Treg ratios of 1/1 or 2/1 mitigated GVHD both in mouse-to-mouse (6–8) and in humanized (9–11) mouse models. These observations prompted pilot trials of adoptive Treg transfer for GVHD prevention in patients given T-cell depleted HLA-haploidentical transplantation (12), as treatment for chronic GVHD (13), and more recently for GVHD prevention in patients given T-cell depleted grafts from HLA-matched donors (14). These trials showed promising results, although the doses of Treg used were high and required an additional day of apheresis collection.

Indeed, an important limitation of Treg adoptive therapy is their low frequency in the peripheral blood and, as a consequence, the limited number of Treg that can be harvested. Administration of low-dose IL-2 has been shown to allow the *in vivo* expansion of infused Treg (15). However, this strategy also expand other cell subsets such as NK cells (16), and may also promote activated (CD25^+^) CD8^+^ T-cells (17). Other strategies aimed at amplifying Treg have been developed and assessed in clinical trials (18, 19). Infusion of third-party expanded Treg in the setting of double umbilical cord blood transplantation resulted in a lower than expected incidence of acute GVHD (18). In contrast, a phase II randomized trial of autologous expanded Treg in children with new-onset type 1 diabetes failed to demonstrate a benefit for Treg infusion (20). Interestingly, in that study, lower rates of *in vitro* expansion during Treg manufacturing significantly correlated with better maintenance of C-peptide 1 year after Treg infusion, strongly suggesting that Treg product properties was a key factor for efficacy.

Given that *ex vivo* manipulation of Treg can alter their suppressive function and *in vivo* persistence, the availability of robust and reliable models to evaluate these parameters is critical. Up to now, the most widely used model consisted of co-transplanting expanded Treg plus human PBMC in NSG mice (10). However, this model is limited by the inability to discriminate between the infused, manipulated Treg and endogenous Treg present within the transferred PBMC population, thereby precluding accurate assessment of the persistence and *in vivo* expansion of manipulated Treg. Another limitation is that this model assesses only the ability of Treg to control xenoreactivity and not alloreactivity. To circumvent these limitations, we developed a novel model in which CD25-depleted PBMC are infused in NSG-HLA-A2-HHD mice (a mouse model in which xeno- and allo-reactivity coexist (21), which we named the “HuCD25neg-PBMC-NSG-Treg model”), followed two days later by Treg infusion. We used this model to evaluate the impact of TNF-α priming on Treg function *in vivo*. Indeed, in mouse-to-mouse models of GVHD, Treg-mediated GVHD protection is dependent on Treg activation by TNF-alpha through the TNFR2 receptor since co-transplantation of TNFR2-deficient Treg fails to attenuate GVHD (22). Furthermore, Pierini et al. demonstrated that TNF-α priming of Treg increased their ability to mitigate GVHD when donor Treg were co-injected at sub-optimal Treg/Tconv ratio (23). However, there are important differences between mouse and human immune systems (24, 25), particularly in regards to Treg biology (1, 26, 27). Moreover, while TNFR2 expression by T-cells is restricted to Treg in mice, human Tconv express high levels of TNFR2 following strong TCR activation *in vitro* (28). These observations prompted us to investigate whether TNF-α priming of Treg would increase their ability to prevent GVHD in the HuCD25neg-PBMC-NSG-Treg model.

As Treg cell-based therapies are under evaluation in a number of diseases, this model could become a tool to improve *ex vivo* manipulating techniques of human Treg prior to clinical trials.

## 2 Materials and methods

### 2.1 PBMC isolation and selection of regulatory T cells for *in vitro* experiments

Prior to *in vivo* testing, the impact of TNF-α on Treg phenotype was assessed *in vitro* as an example of Treg *ex vivo* manipulation technique.

First, PBMC were isolated from buffy coats derived from healthy donors’ blood donations (Belgian Red Cross). They were isolated by density centrifugation with Ficoll^®^ Paque Plus (Merck, Darmstad, Germany). Blood was divided into two tubes with 20 mL in each. Dulbecco’s Phosphate Buffered Saline (PBS) (Biowest, Nuaillé, France) was added in each tube to a total volume of 35 mL (20 mL of blood and 15 mL of PBS). Next, the dilution was carefully overlaid on 15 mL of Ficoll^®^ Paque Plus and centrifuged at 500g with an acceleration of 6 and a deceleration of 4 for 30 minutes at room temperature (RT). Plasma was discarded and the PBMC layer was collected and poured into a new tube. Cells were washed thrice with PBS at 500g for 5 minutes at RT for each wash and then resuspended in 10 mL of PBS to be counted with a Sysmex XS-800i cell counter (Sysmex Europe SE, Norderstedt, Germany).

Secondly, Treg were isolated by immuno-magnetic selection with the EasySep ™ Human CD4^+^CD127^low^CD25^+^ Isolation kit (Stemcell Technologies, Vancouver, Canada). PBMC were resuspended in recommended medium composed of PBS and 2% of Fetal Bovine Serum (FBS) (Gibco™, Thermo Fischer Scientific, Waltham, Massachusetts, USA) at a cell concentration of 50 x 10^6^ PBMC per mL. The protocol from the kit was carefully followed and the EasyEight™ EasySep™ Magnet (Stemcell Technologies) was used for multiple sample immuno-magnetic selection. Using this kit, Treg were isolated with a purity ranging from 75 to 90% (**Supplementary Figure 1**).

Isolated Treg were cultured with TNF-α at different concentrations for 48 hours. Eighty thousands Treg per well were plated in a 96-well U bottom plate with either recombinant human IL-2 at 10 ng/mL alone, IL-2 and TNF-α both at 10 ng/mL, or IL-2 at 10 ng/mL and TNF-α at 100 ng/mL. Each condition was divided in two: half the cells were not exposed to beads (non-activated cells) and the other half was exposed to anti-human CD3/CD28 beads (Dynabeads™ Human T-Activator CD3/CD28 for T Cell Expansion and Activation, Gibco™) at a bead-to-cell ratio of 1:4. The purpose of using beads was to put Treg in conditions comparable to the *in vivo* setting, as it has been demonstrated that the CD3/CD28 pathway is activated in GVHD (21). Cells were incubated at 37°C in 5% CO2.

Culture medium was composed of RPMI 1640, human serum, glutamine and penicillin-streptomycine. Cytokines (IL-2 and TNF-α) were purchased from Peprotech (nowadays part of Thermo Fischer Scientific). A list of materials used in cell culture is detailed in **Supplementary Table 1**.

### 2.2 Spectral flow cytometry for *in vitro* experiments

After 48 hours of incubation, cells were collected, washed with 1 mL of PBS at 500g for 5 minutes (of note, centrifugation parameters for washes were always 500g for 5 minutes) and counted before being stained with a panel designed for spectral flow cytometry, including 22 antibodies (**Supplementary Table 2**). First, cells were incubated with a Fc blocker to prevent unspecific antibody binding. Two µL of Fc blocker mixed with 48 µL of staining buffer were deposited on cell pellets for 10 minutes in the dark at RT. Samples were washed with 1 mL of staining buffer (PBS with 3% of FBS) and incubated with the first mix of extracellular antibodies for 20 minutes at 4°C in the dark.

Cells were washed with 1 mL of staining buffer, stained with the second mix of extracellular antibodies and incubated for 20 minutes at 4°C in the dark. Extracellular antibodies were divided into two mixes to prevent steric hindrance. Cells were washed with 1 mL of PBS and incubated with 200 µL of Fixable Viability Dye efluor™ 780 diluted at 1:1000 in PBS for 30 minutes at 4°C in the dark. Cells were washed with 1 mL of staining buffer before being permeabilized using the FOXP3/Transcription Factor Staining Buffer Set (Invitrogen™, Thermo Fischer Scientific) with an incubation of 30 minutes in the dark. After two washes with the washing buffer from the kit (one of 500 µL and one of 1 mL), cells were stained with the mix of intracellular antibodies. Cells were washed once with 2 mL of the washing solution, then once with 1 mL of PBS and resuspended in 200 µL of PBS. Cells were then ready to be analyzed.

Data were acquired on a 5-laser (355 nm, 405 nm, 488 nm, 561 nm, 633 nm) SONY ID7000 Spectral Cell Analyzer (Sony Biotechnology, San Jose, California, USA) and analyzed with FlowJo Software v10.8.1 (Tree Star Inc., Ashland, Oregon, USA) for supervised gating/population definitions. Gating strategy is shown in **Supplementary Figure 2**. Of note, fluorescence minus one (FMO) controls were used to ensure accurate gate placement (data not shown).

Three independent experiments were performed with two blood donors for each, for a total of 6 donors.

### 2.3 Unsupervised spectral flow cytometry analyses for *in vitro* experiments

Prior to analysis, we worked on the FlowJo software v10.8.1 (Tree Star Inc.) to scale the fluorescence of each marker in order to optimize data visualization and clustering. Analyses were made on Treg. Data was exported as CSV files in R software (version 4.3.1) for each condition. For experiments without CD3/CD28 beads, 14077 Treg for each donor were put in the dataset. For experiments with CD3/CD28 beads, 13488 Treg for each donor were put in the dataset. Data was analyzed through successive FlowSOM clustering (29) and t-distributed stochastic neighbor embedding (t-SNE) representation after exporting similar event numbers for each sample per condition group, using an in-house script developed by Rocha et al. (30), as previously reported (30–32). FlowSOM t-SNE and UMAP clusters were formed based on a list of markers (GARP, CD127, CD25, ICAM-1, TIGIT, HLA-DR, CTLA4, PD-1, ICOS, TIM3, CD45RA, LAP, TNFR2, CD26, HELIOS, CD39, CD45RO, CD69, LAG3 and FOXP3). Differences between t-SNE representations were calculated following the same approach as in the t-SNE algorithm. For t-SNE, datasets were analyzed with 5,000 iterations and perplexity of 30. Then, all pairwise comparisons between plots were evaluated with Kolmogorov–Smirnov tests on the differences between the cross-entropy distributions. Resulting p values were corrected with the Holm method. Dendrograms were obtained from hierarchical clustering, using the Kolmogorov–Smirnov statistic as a distance measurement.

### 2.4 Single cell RNA sequencing

Single cell RNA sequencing was performed in order to characterize the impact of TNF-α priming on transcriptomics in Treg. PBMC from three healthy donors (Belgian Red Cross) were used for that experiment. Treg were isolated as previously described and cultured for 24 hours under two conditions: Treg with IL-2 at 10 ng/mL alone or Treg with IL-2 at 10 ng/mL and TNF-α at 100 ng/mL. Cell concentration was 80,000 cells per 200 µL in a 96-well U bottom plate. After 24 hours, cells were collected and washed with 1 mL of PBS. We worked with the GIGA Genomics Platform (https://www.gigagenomics.uliege.be/cms/c_4164592/en/gigagenomics) to prepare cells for single cell RNA sequencing. The 10x Genomics 3’ CellPlex Kit (10x Genomics B.V., Leiden, the Netherlands) was used to perform cell multiplexing: our six samples were labelled individually with cell multiplexing oligonucleotides. This allowed us to pool samples and read them together.

Materials used for single cell RNA sequencing are listed in **Supplementary Table 3**.

#### 2.4.1 Cell multiplexing

As described in the kit protocol, 1 mL of PBS with BSA 0.04% (Sigma-Aldrich, Merck KGaA, Darmstadt, Germany) were added to each sample (approximately 1 million cells per sample) which were then centrifuged at 400g for 5 minutes at RT. The supernatant was removed and cells were resuspended in 100 µL of Cell Multiplexing Oligo (one oligonucleotide per sample, 3’ CellPlex Kit Set A, 10x Genomics B.V.) by gently pipetting 10–15 times. Cells were incubated at RT for 5 minutes and mixed by gently pipetting with 1.9 mL of Wash and Resuspension buffer (composed of PBS and BSA 1%). Cells were centrifuged at 400g for 5 minutes at 4°C, the supernatant was removed, and two additional washes were made with 2 mL of the Wash and Resuspension Buffer (total of three washes). The supernatant was removed and cells were resuspended in the Wash and Resuspension Buffer to be counted (volume according to protocol) and assessed for viability using a Countness™ 2 Automated Cell Counter (Thermo Fischer Scientific). Next, labeled cells were pooled and the number of cells in the final mixture was counted.

#### 2.4.2 Library preparation (performed by the GIGA Genomics Platform)

A total of 20,000 equimolarly pooled, CellPlex-labeled cells were processed using the Chromium Next GEM Single Cell 3′ Reagent Kit v3.1 with Cell Multiplexing (10x Genomics, protocol CG000390) on a Chromium iX instrument with Chip G (10x Genomics).

##### 2.4.2.1 Single-cell Gene Expression Matrices (GEMs) generation

GEMs were generated by combining barcoded Single Cell 3′ v3.1 Gel Beads, a Master Mix, and CellPlex-labeled cells with Partitioning Oil on a Chromium Chip G (10x Genomics). Reverse transcription was performed using a Veriti™ Thermal Cycler (Thermo Fisher Scientific) with the following conditions: 53°C for 45 minutes, 85°C for 5 minutes, and holding at 4°C.

##### 2.4.2.2 cDNA amplification and purification

Following emulsion breakage, pooled fractions were purified using DynaBeads MyOne Silane Beads (10x Genomics). cDNA and CellPlex barcodes were amplified via PCR with a 12-cycle reaction module: 98°C for 3 minutes, 98°C for 15 seconds, 63°C for 20 seconds and 72°C for 1 minute, followed by a final extension at 72°C for 1 minute. Feature cDNA Primer 3 was used to amplify both cDNA and CellPlex (primer included in the kit).

The cDNA reaction was purified using magnetic SPRI Beads (Beckman Coulter, Indiana, United States). The supernatant, containing barcoded CellPlex amplicons, was separated and purified again for CellPlex library preparation.

##### 2.4.2.3 Library construction for 3’ gene expression

For 3′ Gene Expression libraries, amplified cDNA was fragmented, end-repaired, A-tailed and ligated to sequencing adapters. Libraries were then amplified by PCR (indexing PCR) using Dual Index TT primers plate (10x Genomics).

##### 2.4.2.4 CellPlex library construction

Purified CellPlex amplicons were processed in an indexing PCR using Feature SI Primers 2 (included in the kit) with dual single index primers (Dual Index Kit NN Set A, 10x Genomics).

##### 2.4.2.5 Library quality control and sequencing

Both the 3′ Gene Expression and CellPlex libraries were quality-checked on QIAxcel Advanced System (QIAGEN, Hilden, Germany) using a DNA screening cartridge. Final dual-indexed libraries were quantified by qPCR with KAPA Library Quantification Kit including Illumina standard (Roche, Basel, Switzerland).

Libraries were sequenced on an Illumina NovaSeq 6000 (Illumina, San Diego, California, USA) using a S4 flow cell for paired-end 150 cycles, and demultiplexed with read 1: 28 cycles; read 2: 91 cycles; index i7: 10 cycles; index i5:10 cycles. Sequencing depth was set to ~20,000 reads per cell for gene expression libraries and 5,000 reads per cell for CellPlex libraries.

##### 2.4.2.6 Raw data preparation

Raw sequencing data were demultiplexed using sample-specific indexes, filtered for quality, and converted into FASTQ files for downstream analysis, using the bcl2fastq software (Illumina).

Sequencing data quality was assessed using the FastQC tool (Illumina) for individual reports per sample, and with MultiQC (Illumina) to generate a report comprising all samples.

#### 2.4.3 Pre-processing and quality control of single-cell RNA-sequencing data

Raw reads from single cell RNA sequencing were demultiplexed and mapped to the GRCh38 human reference genome using CellRanger software (v. 7.1.0, 10x Genomics B.V.) with default parameters. The following tags (Combined Lineage Tracing, CMO) were associated with each sample: CMO307 for donor 3 with TNF (3-EXP), CMO308 for donor 3 without TNF (3-CTRL), CMO309 for donor 4 with TNF (4-EXP), CMO310 for donor 4 without TNF (4-CTRL), CMO311 for donor 7 with TNF (7-EXP), CMO312 for donor 7 without TNF (7-CTRL). Cells were selected based on three criteria (i) the number of reads sequenced (threshold = 1,000) (ii) the number of expressed genes (threshold = 600) and (iii) the percentage of mitochondrial genes (threshold = 10%). These thresholds were established based on parameter distribution. After this filtration step, we obtained 7048 good quality cells (starting from 7372).

#### 2.4.4 Dimensionality reduction and cell clustering

The scanpy (sc) package (v. 1.9.3) implemented in Python software v.3.10.9 (Python Software Foundation, Beaverton, Oregon, USA) was used to perform the dimensional reduction of Treg. To normalize raw data, we used the “sc.pp.normalize_total” function, specifying that we wanted a total of 10,000 Counts Per Million (CPM). The “sc.pp.highly_variable_genes” function was used to extract the most variable genes for each sample. We then calculated clusters using the Leiden algorithm by the “sc.tl.leiden” function, with resolutions between 0.4 and 0.8, and integrated with the UMAP algorithm “sc.tl.umap” with the following parameters: min_dist=0.5, gamma=1, alpha=1, negative_sample_rate=5, init_pos=“spectral”.

#### 2.4.5 Isolation of CD4^+^ CD25^+^ cells

We first applied the batch effect in order to get rid of the donor effect (each donor was considered as a separate batch) using the Harmony method “sc.external.pp.harmony_integrate”. Secondly, we selected Treg from our raw data based on the expression of gene markers “CD4” and “IL2RA”.

#### 2.4.6 Differential gene expression

Differentially expressed genes (DEGs) for each cluster were identified using the “sc.tl.rank_genes_groups” function with the Student’s t-test to evaluate statistical significance. Genes were classified as significant DEGs if they had an adjusted p-value (FDR) < 0.05 and an absolute value of log fold-change (FC) > 1.

#### 2.4.7 Functional enrichment analysis of signature genes

Gene set enrichment analysis (GSEA) was conducted using the “gp.prerank” function from the Gseapy package, with Hallmark gene sets obtained from the MSigDB website (gsea-msigdb.org). Heatmaps were generated with the “sc.pl.heatmap” function to visualize gene expression across conditions within the most relevant pathways.

### 2.5 Treg activation by TNF-α for the *in vivo* experiment

As described above, PBMC were isolated from buffy coats by density centrifugation. During this process, staining with an anti-human HLA-A2 antibody was performed on blood from the different buffy coats after red blood cell lysis in order to select HLA-A2 negative donors. Indeed, in this model, the advantage of selecting a HLA-A2 negative donor is to obtain an allogeneic response as these mice express the human HLA-A2 antigen.

Secondly, PBMC were CD25-depleted and human Treg were sorted using immuno-magnetic selection with the EasySep ™ Human CD4^+^CD127^low^CD25^+^ Isolation kit. The protocol of the kit was modified to allow both CD25 depletion and Treg isolation. The following steps were taken: 1) Addition of CD25-Positive Selection Cocktail at a volume of 50 µL/mL of sample, 2) Vortex and incubation for 5 minutes at RT, 3) Vortex Releasable Rapidspheres for 30 seconds 4) Addition of Release Rapidspheres at a volume of 30 µL/mL of sample 5) Instead of adding the CD4+ T-cell enrichment cocktail to the sample, cells were incubated for 5 minutes (**protocol modification**) 6) Samples were topped up with recommended medium to 5 mL (if they were < 4 mL) or to 10 mL (if they were > 4 mL) then placed on the EasyEight™ Easysep™ magnet for 10 minutes 7) The supernatant, containing a solution depleted in CD25^+^ cells, was collected and placed in new tubes for a second incubation of 5 minutes 8) The supernatant was collected and centrifugated at 500g for 5 minutes at RT. The pellet was resuspended in 10 mL of PBS and CD25-depleted PBMC were counted on the Sysmex cell counter. These cells were used for mouse transplantation and the purity was assessed by conventional flow cytometry (**Supplementary Figure 3**). The panel used for cell purity is detailed in **Supplementary Table 4**.

Next, Treg were isolated from the CD25^+^ enriched fraction which had been retained on the walls of the tubes by the magnetic reaction after step 6. Tubes were topped up with recommended medium to 5 or 10 mL (depending on the initial volume of each sample). The following steps were then carried out: 1) Tubes were placed on the magnet for 5 minutes 2) Supernatant was discarded then samples were topped up to 5 or 10 mL 3) Tubes were placed on the magnet for another incubation of 5 minutes 4) Supernatant was discarded 5) Tubes were filled with recommended medium at the same volume as the original sampling volume 6) Addition of release buffer at a volume of 100 µL/mL of sample (to release Rapidspheres) 7) Samples were mixed by pipetting up and down more than 5 times 8) CD4^+^ T-cell enrichment cocktail was added at a volume of 50 µL/mL of sample and CD127high depletion cocktail was added at a volume of 50 µL/mL of sample (**modification from the initial protocol**) 9) Cells were incubated for 5 minutes at RT 10) Dextran Rapidspheres were vortexed for 30 seconds and added at a volume of 10 µL/mL of sample 11) Samples were mixed and incubated at RT for 5 minutes 12) Samples were topped up to 5 or 10 mL with recommended medium and then placed onto the magnet for a 5-minutes incubation 13) The supernatant, enriched in CD25^+^CD127^low^CD4^+^ T-cells, was collected and washed. The pellet was resuspended in 2 mL of culture medium. The purity of Treg was similar with this protocol modification compared to the initial protocol (purity of 75-90%).

After immuno-magnetic selection, Treg were counted with the Sysmex cell counter and culture medium was added to obtain the wanted cell concentration (500,000 per mL). Cells were divided into two tubes: in the first tube, IL-2 only was added at the concentration of 10 ng/mL while in the second tube, IL-2 at 10 ng/mL and TNF-α at 100 ng/mL were added. Cells were seeded in a 96-well round-bottom plate at the concentration of 100,000 cells in 200 µL of culture medium per well. Cells were then incubated for 48 hours at 37°C in 5% CO2.

### 2.6 Induction and assessment of xGVHD in NSG-HLA-A2/HHD mice

#### 2.6.1 Mice

NSG-HLA-A2/HHD mice were purchased from The Jackson Laboratory (Bar Harbor, Maine, USA) and bred in house at the University of Liège (ULiège), Belgium. Mice were housed in pathogen-free conditions in microisolator cages and fed standard rodent chow diet. Female and male mice aged between 8 and 12 weeks were used in each experiment. Mice were euthanized with an intraperitoneal injection of 1520mg/kg of pentobarbital and 8mg/kg of lidocaine chloride (i.e. 200µl of pentobarbital 200mg/ml (Dolethal™, Vetoquinol, Niel, Belgium) mixed with 5% of lidocaine chloride 20mg/ml (Xylocaine ^®^, Aspen Pharma Trading Limited, Dublin, Ireland), for an average mouse weight of 25g), followed by cervical dislocation 15 minutes after the injection and only in the absence of breathing signs. The method for euthanasia was recommended by the Institutional Animal Care and Use Ethics Committee of the University of Liège, Belgium.

All animal experiments used in this study were reviewed and approved by the Institutional Animal Care and Use Ethics Committee of the University of Liège (file # 20-2262). The “Guide for the Care and Use of Laboratory Animals,” prepared by the Institute of Laboratory Animal Resources, National Research Council, and published by the National Academy Press, was followed cautiously.

#### 2.6.2 Transplantation and monitoring

NSG-HLA-A2/HHD, aged 8 to 12 weeks, were irradiated with 2 Gy the day prior to transplantation. On day 0, they were infused with 2x10^6^ CD25-depleted hPBMCs from non-HLA-A2 donors to induce x/aGVHD. Nine PBMC donors were used for three independent experiments (4 donors in the first experiment, 3 donors in the second and 2 donors in the third). For each PBMC donor, 2x10^6^ CD25-depleted hPBMCs were injected into mice while Treg were cultured under two conditions: IL-2 at 10 ng/mL alone or IL-2 at 10 ng/mL and TNF-α at 100ng/mL. After 48 hours of incubation, Treg were collected, washed and counted with the Sysmex cell counter. Treg were resuspended in PBS at a concentration of 0.5 x 10^6^ in 200 µL (2.5 x 10^6^ per mL). For each PBMC donor, mice were divided into three groups: the control group did not receive any Treg, the unprimed group received on day 2 0.5x10^6^ Treg incubated with IL-2 alone and the primed group received on day 2 0.5x10^6^ Treg incubated with IL-2 and TNF-α. A total of twenty-seven mice were used, with nine mice in each group (4 males and 5 females in each group, for a total of 12 males and 15 females). Males and females were used in all three experiments with a distribution of sex as follow: 5 males and 7 females in the first experiment, 3 males and 6 females in the second and 4 males and 2 females in the third.

Peripheral blood (PB) samples were collected on days 14, 21 and 28 after PBMC infusion for quantification of human immune cell engraftment by flow cytometry (see below). GVHD severity was assessed by a scoring system that incorporates four clinical parameters: weight loss, posture (hunching), mobility and anemia, as previously reported (33). Each parameter received a score of 0 (minimum) to 2 (maximum). Mice were assessed for GVHD score thrice weekly and monitored daily. Mice reaching a GVHD score of 6/8 were sacrificed in agreement with the request of our ethical committee. Final scores for dead animals reaching the ethical limit score were “6” for the remaining time points. Final weights for dead animals reaching the ethical limit score were kept in the data set for the remaining time points (last value carried forward). After sacrifice, lungs and liver were collected and embedded in paraffin for further investigations. No blinding was done regarding treatment received but GVHD scorings were performed by a technician (SD) who is in charge of mouse welfare and not interested in the study outcome.

#### 2.6.3 Flow cytometry for *in vivo* experiments

PB was drawn from mouse tails on days 14, 21 and 28 and depleted of erythrocytes using Red Blood Cell lysis buffer (eBioscience™, Thermo Fischer Scientific) to retain only white blood cells. Two mL of lysis buffer (previously diluted at 1:10 in distilled water) were added to 150 µL of blood and the solution was incubated at RT for 5 minutes. Cells were washed twice with 2 mL of staining buffer. Of note, all centrifugations described for washes were made at 500g for 5 minutes at RT.

Staining for conventional flow cytometry was performed with a 13-color panel, designed to assess immune reconstitution. The composition of the panel is detailed in **Supplementary Table 5**, with antibodies targeting antigens expressed on human white blood cells and one antibody targeting mouse CD45. First, samples were incubated with 50 µL of the mix of extracellular antibodies for 20 minutes in the dark at 4°C and then washed once with 1 mL of PBS. Next, cells were incubated for 30 minutes at 4°C in the dark with a Fixable Viability Dye efluor™ 780 and washed once with 1 mL of staining buffer. Cells were permeabilized using the FOXP3/Transcription Factor Staining Buffer Set (Invitrogen™, Thermo Fischer Scientific) with an incubation of 30 minutes at 4°C in the dark. Cells were washed twice with the washing buffer from the kit (once with 500 µL and once with 1 mL) and incubated with 50 µL from the mix of intracellular antibodies for 30 minutes at 4°C in the dark. Cells were washed with 2 mL of washing buffer, then 1 mL of PBS, and resuspended in 200 µL of PBS.

Data were acquired on a BD LSR FORTESSA Flow Cytometer (BD Biosciences, Becton Dickinson, Franklin Lakes, New Jersey, USA) and analyzed with the FlowJo Software v10.8.1 (Tree Star Inc.). In the gating strategy, Treg were defined as CD4^+^CD25^+^CD127^low^FOXP3^+^ while the remaining CD4^+^ T-cells were termed conventional T cells (Tconv). Total cell counts of human CD45^+^ cells in blood were calculated based on the absolute number of white blood cells (counted with an ABX Diagnostics Micros 60 (AxonLab)) and on human cell chimerism (frequency of human CD45^+^ cells among the total white blood cell population, calculated as $(\frac{\%human\;CD45+\text{cells}}{\%human\;CD45+\;cells\;+\;\%mouse\;CD45+cells})x100$).

### 2.7 Statistical analyses

The Wilcoxon matched-pairs signed rank test was used to compare flow-cytometry data between two groups (comparison control-unprimed, control-primed, unprimed-primed), both for *in vitro* and *in vivo* experiments. Comparisons between GVHD score and weight loss curves were made using the 2-way ANOVA test. Survival curves were modelled using the Kaplan-Meier method. *P* values < 0.05 were considered statistically significant and all *P* values were 2-sided. Statistical analyses were carried out with Graphpad Prism 8.3.0 (Graphpad Software, San Diego, CA, USA).

## 3 Results

### 3.1 Impact of TNF-α priming on Treg *in vitro*

#### 3.1.1 Spectral flow cytometry

We assessed the impact of TNF-α priming for 48 hours on Treg phenotype using spectral flow cytometry. These experiments were performed with and without stimulation of Treg by CD3/CD28 beads since we previously demonstrated that these pathways are activated in human T-cells once injected in NSG or NSG-HLA-A2-HHD mice (21). Furthermore, Treg stimulated with CD3/CD28 beads express more TNFR2 and could better respond to TNF-α priming.

**Figure 1:**
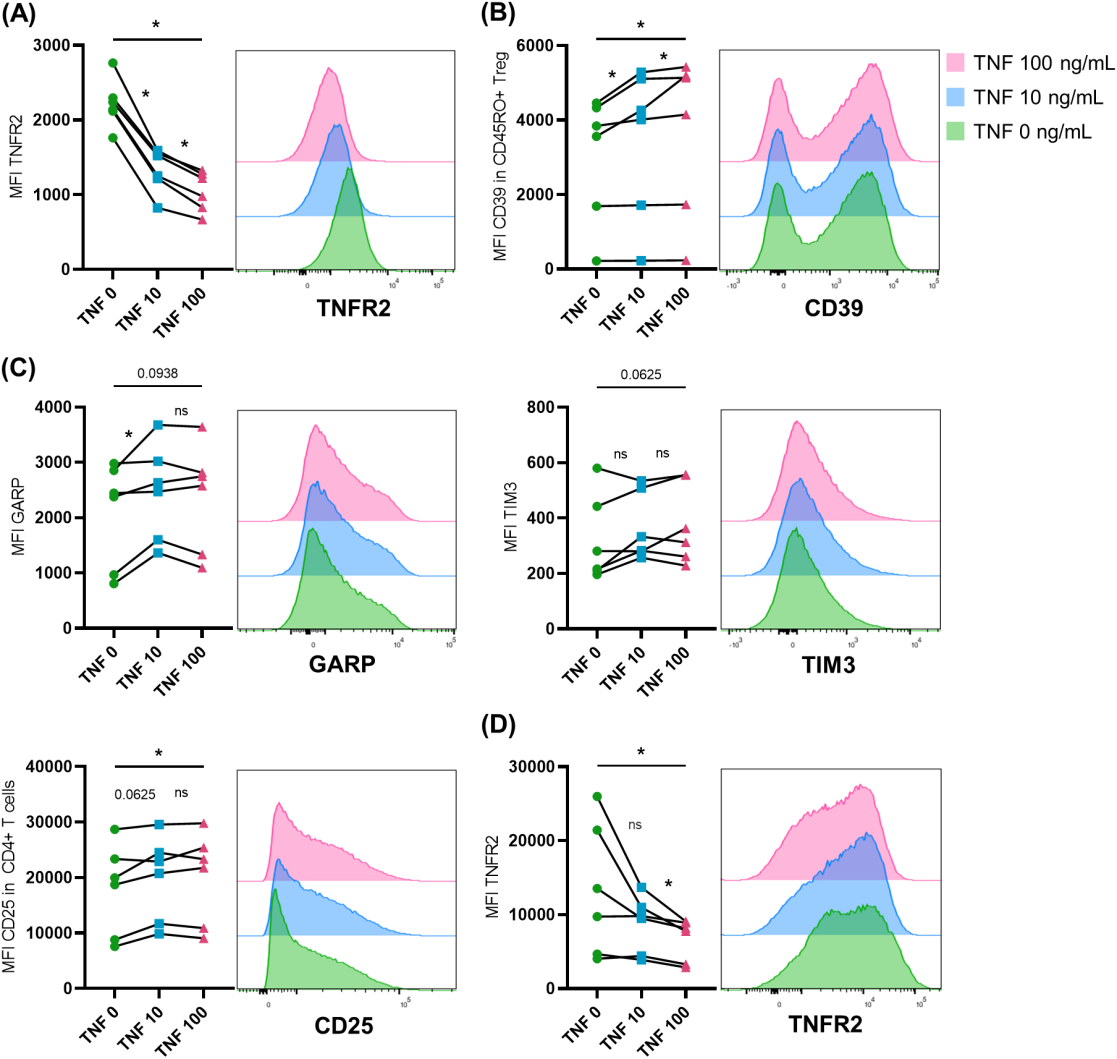
TNF-α priming of human Treg increases their activation (Spectral flow cytometry). Data were acquired from 6 donors in 3 independent experiments. Samples are paired as cells from each donor were divided into 3 conditions: control (no TNF-α), TNF-α at 10 ng/mL and TNF-α at 100 ng/mL. Statistical analyses were performed with a Wilcoxon matched-pairs signed rank test (*p<0.05, **p<0.01) comparing two groups at a time. TNF-α is expressed in ng/mL. Histograms show the mean fluorescence intensity (MFI) for each marker (Y axis = Unit Area), in concatenated data. **(A)** In Treg not stimulated with CD3/CD28 beads, TNFR2 expression decreased after exposure to TNF-α. **(B)** In Treg not stimulated with CD3/CD28 beads, an increased expression of CD39 was observed in CD45RO^+^ Treg. **(C)** In Treg stimulated with CD3/CD28 beads, the expression of GARP, TIM3 and CD25 was increased upon TNF-α exposure. **(D)** In Treg stimulated with CD3/CD28 beads, the expression of TNFR2 was also decreased when Treg were exposed to TNF-α.

Using conventional gating (**Supplementary Figure 2**), we observed that TNF-α priming of non-stimulated Treg decreased their TNFR2 expression while increasing CD39 expression by CD45RO^+^ Treg (p = 0.03) (**Figures 1A, B**). Analyzing data using t-SNE/FlowSOM clustering, asymptotic two-sample Kolmogorov-Smirnov test demonstrated that TNF-α priming significantly altered the t-SNE distribution (p=2.16x10^-9^) (**Supplementary Figure 4**).

As expected, stimulation with CD3/CD28 beads significantly impacted Treg phenotype, inducing an increased expression of most markers (**Supplementary Figures 5, 6**). TNF-α priming of Treg in the presence of CD3/CD28 beads resulted in higher expression of GARP (p = 0.03 and p = 0.09 at 10 ng/mL and 100 ng/mL, respectively), TIM-3 (p = 0.06 at 100 ng/mL) and CD25 (p = 0.06 and p = 0.03 at 10 ng/mL and 100 ng/mL, respectively) (**Figure 1C**). In contrast, as observed without microbeads, TNFR2 expression significantly decreased following TNF-α priming (**Figure 1D**).

We next analyzed data using t-SNE/FlowSOM clustering and the asymptotic two-sample Kolmogorov-Smirnov test confirmed that TNF-α priming significantly altered the t-SNE distribution (p=1.207x10^-11^) in Treg stimulated with CD3/CD28 beads (**Supplementary Figure 7**). We observed that 100 ng/mL TNF-α priming induced a higher proportion of Treg among cluster #2 (memory Treg CD26^neg^CD39^+^CD69^+^ICOS^+^, corresponding to the most differentiated Treg fraction expressing ICOS and CD69, p = 0.031) while in contrast there was a suggestion for a lower proportion of Treg in cluster #4 (memory Treg CD26^neg^CD39^+^CD69^neg^ICOS^neg^, corresponding to the most differentiated Treg fraction not expressing ICOS and CD69, p = 0.094).

Of note, unsupervised analysis also emphasized an increased expression of activation markers after TNF-α exposure in Treg activated with CD3/CD28 beads such as GARP (p = 0.031), CD25 (p = 0.036), TIGIT (p = 0.059) and TIM3 (p = 0.062). In Treg not exposed to CD3/CD28 beads, TNF-α tended to increase the expression of ICAM (p = 0.036), TIM3 (p = 0.058), TIGIT (p = 0.058), CD69 (p = 0.058), CD26 (p = 0.059) and FOXP3 (p = 0.062) (**Supplementary Figure 8**).

Taken together, these data show that, following TNF-α priming, several markers associated with higher Treg activity, such as CD39 (when no beads were added) or GARP (when CD3/CD28 beads were added), were overexpressed.

#### 3.1.2 Single cell RNA sequencing

We assessed the impact of 24-hour TNF-α priming on Treg transcriptomics with single cell RNA sequencing. Using a resolution of 0.30, we observed 5 Treg clusters (**Supplementary Figure 9A**). Cluster # 4 included almost only TNF-α primed Treg, while clusters #0, #1, #2 and #3 included both TNF-α activated and control Treg (**Supplementary Figure 9B**). Among clusters #0-#3, clusters 2 and 3 included Treg with a more naive phenotype (CCR7 and to a lesser extent DPP4 (coding for CD26) positive, as well as lower expression of IL2RA (coding for CD25) and FOXP3) while clusters 0 and 1 included memory Treg (CCR7 negative and ENTPD1 (coding for CD39) positive, with a higher expression of IL2RA and FOXP3). Cluster #4 was CCR7^+^ but harbored a more active phenotype with a higher expression of IL2RA (coding for CD25), FOXP3, ICOS and TIGIT (**Supplementary Figure 9C**).

Differential expression analysis revealed that TNF-α primed Treg exhibited elevated expression of key markers, including IL2RA (coding for CD25), IL-32, TNFRSF18 (coding for GITR) and TNFRSF4 (coding for OX40) (**Supplementary Figure 9D**), with fold changes of 2.5 for IL2RA (adjusted p = 3.35 × 10^15^), 10 for IL-32 (adjusted p = 7.00 × 10^141^), 66 for TNFRSF18 (adjusted p = 2.82 × 10^122^), and 309 for TNFRSF4 (adjusted p = 1.50 × 10^270^). FOXP3 expression showed a modest increase (fold change = 1.38), but this did not reach statistical significance (adjusted p = 0.08).

We next investigated which genes were upregulated in Treg following TNF-α priming (**Supplementary Figure 10A**). Using GSEA analyses in the whole Treg population, we observed that 11 Hallmark pathways were significantly upregulated following TNF-α priming. These pathways included interferon-gamma response, TNF-α signaling via NF-kB, interferon-alpha response, IL6-JAK-STAT3 signaling, inflammatory response, oxidative phosphorylation, allograft rejection, UV response UP, MTORC1 signaling, apoptosis and IL2-STAT5 signaling (**Supplementary Figure 10B**). We then investigated whether similar pathways were activated by TNF-α in naive and memory Treg subsets. We observed that seven Hallmark pathways were commonly upregulated by TNF-α priming in both Treg subsets (**Supplementary Figures 10C, D**). In addition, in naive Treg, other signaling pathways, such as oxidative phosphorylation and MTORC1 signaling were activated when Treg were primed with TNF-α. Taken together, these data show that TNF-α priming activates both naive and memory Treg subsets.

### 3.2 The HuCD25^neg^-PBMC-NSG-Treg model provides *in vivo* assessment of infused Treg

2 Gy irradiated NSG-HLA-A2/HHD mice (n=3/PBMC donor) were i.v. infused with 2x10^6^ CD25-depleted human PBMC/mouse. One mouse/PBMC donor served as control, the two remaining mice received 0.5 x 10^6^ non-primed (n=1) or TNF-α primed (n=1) Treg/mouse, two days later. The experiments were performed with a total of 9 PBMC donors (total of 27 mice, 9 mice per group).

#### 3.2.1 Infused Treg engraft in HuCD25^neg^-PBMC-NSG mice

**Figure 2:**
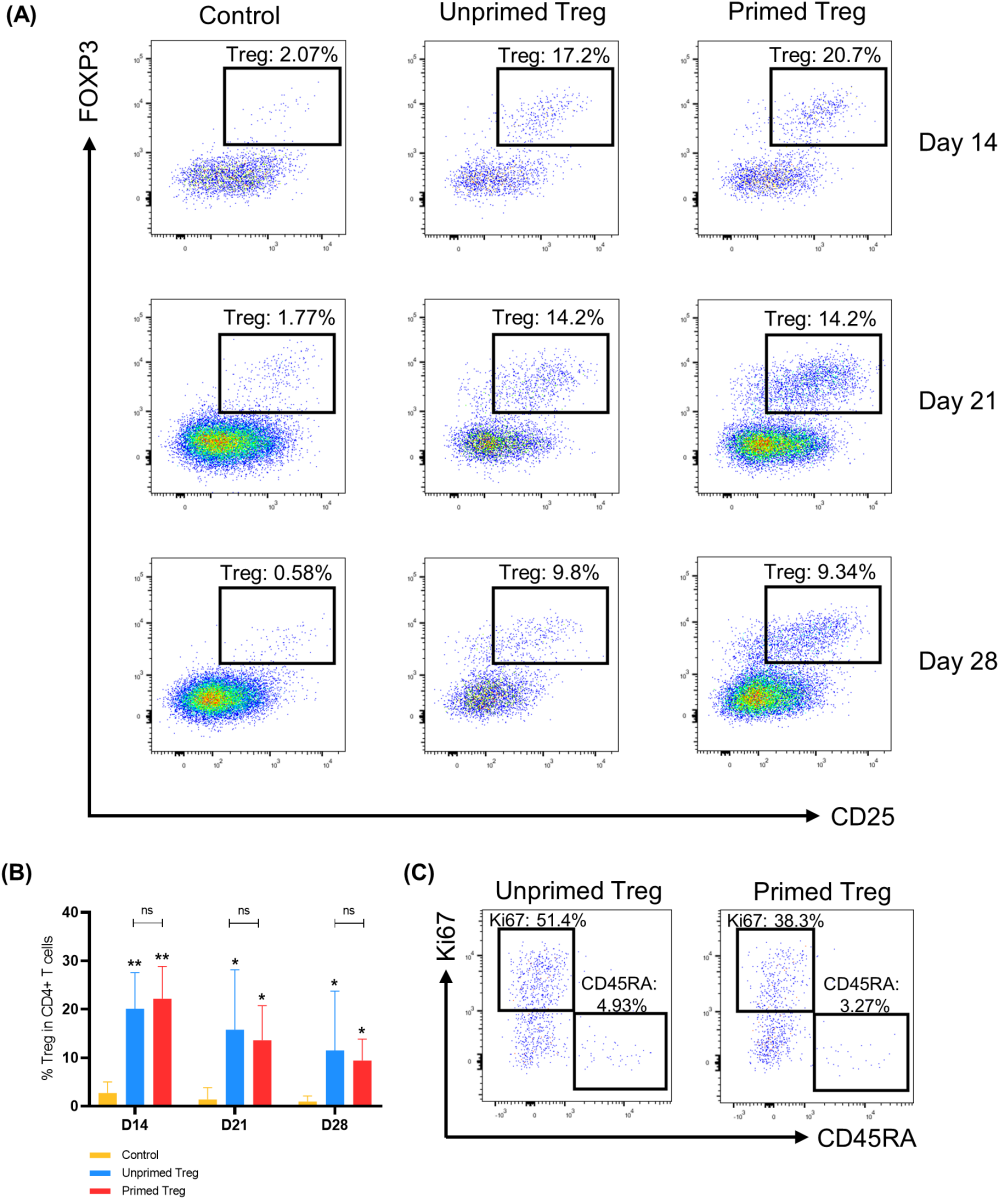
Infused Treg engraft in HuCD25^neg^-PBMC-NSG mice. **(A)** Concatenated data on blood samples at days 14, 21 and 28 for 8 cohorts (cohort 7 was withdrawn because of a very low engraftment). Treg are shown among CD4 ^+^ T-cells. Treg frequencies remained low in control mice which received only CD25-depleted PBMC (n=8 at day 14, n=7 at day 21, n=6 at day 28). In contrast, Treg frequencies were significantly higher in mice that received Treg which had not been cultured with TNF-α (Unprimed Treg, n=8 at all time-points) and in mice that received Treg previously primed with TNF-α (Primed Treg, n=8 at all time-points). **(B)** Treg frequencies among CD4^+^ T-cells at days 14, 21 and 28 after PBMC infusion. The p value above each column is the result of the comparison between the control group and the indicated group with a Wilcoxon matched-pairs signed rank test (*p<0.05, **p<0.01). The percentage of Treg was significantly higher in mice which had received Treg. There was no difference between the unprimed and the primed group. **(C)** Concatenated data on blood samples at day 21 in mice that received unprimed Treg (n=8) and in mice that received primed Treg (n=8). In this model, the persistence of Treg allows their characterization.

We first compared Treg engraftment in the three groups of mice. We observed very few Treg in the peripheral blood of control mice transplanted with CD25-depleted PBMC, demonstrating the efficacy of the depletion and that few (if any) Tconv converted to Treg *in vivo* (**Figure 2A**). As hypothesized, human Treg percentages were significantly higher in the peripheral blood of Treg-infused mice on days 14, 21 and 28 after transplantation, demonstrating that the infused Treg significantly engrafted (**Figure 2B**). The high percentages of Treg in Treg-infused mice enabled the analysis of their phenotype (**Figure 2C**). Interestingly, Treg percentages in mice given non-primed or TNF-α primed Treg were similar, suggesting a lack of benefit of TNF-α priming in this model.

#### 3.2.2 Infused Treg decrease Tconv and CD8^+^ T-cell expansion in HuCD25^neg^-PBMC-NSG mice

**Figure 3:**
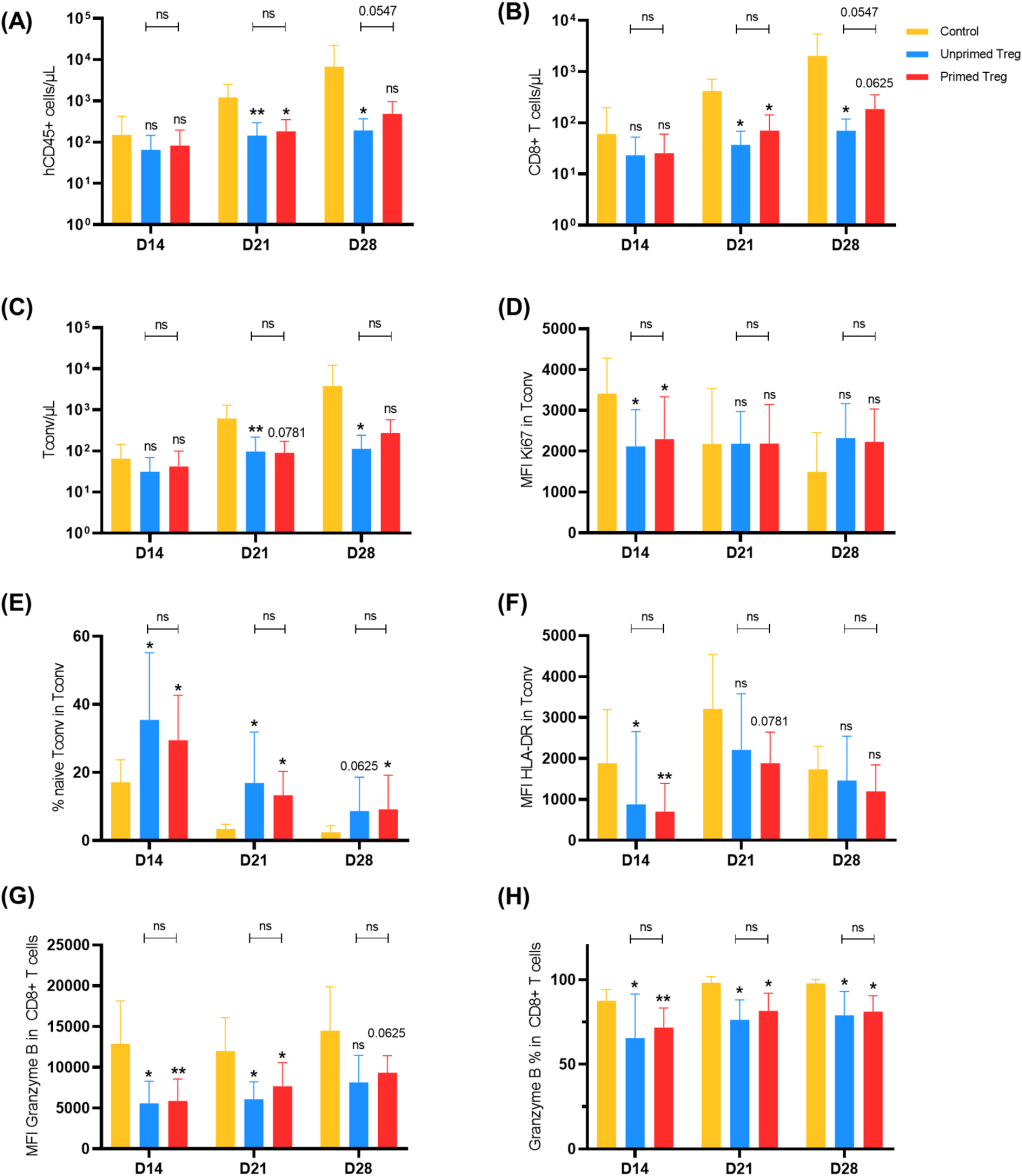
Treg significantly decreased the proliferation and activation of Tconv (CD4^+^CD25^-^ T-cells) and CD8^+^ T-cells *in vivo*. NSG-HLA-A2/HHD mice were transplanted with CD25-depleted PBMC; 48 hours later, mice received PBS only (control), unprimed Treg or primed Treg. Blood samples were drawn on days 14, 21 and 28 following transplantation and stained for flow cytometry. The number of subjects in the control group was 9 at day 14, 8 at day 21 and 7 at day 28. The number of subjects in the unprimed Treg and in the primed Treg groups was 9 in each group at all time points. The results from Cohort 7 were used only for hCD45^+^ count because the engraftment was very low in this cohort. Subsequently, there was one mouse retrieved from each group at each time point for this cohort, expect for hCD45^+^ count. The p value above each column is the result of the comparison between the control group and the indicated group with a Wilcoxon matched-pairs signed rank test (*p<0.05, **p<0.01). **(A)** Human chimerism (number of human CD45^+^ cells/[number of human CD45^+^ cells + number of mouse CD45^+^ cells]x100) was decreased in mice that received Treg. **(B)** Absolute number of CD8^+^ T-cells **(C)** Absolute number of Tconv **(D)** The expression of Ki67 in Tconv was lower at day 14 in mice that received Treg **(E)** The proportion of Tconv with a naive phenotype (CD45RA^+^CD27^+^ Tconv) was significantly higher in mice that received Treg at all time points. **(F)** In mice that received Treg, Tconv harbored a less activated phenotype at day 14. **(G)** The expression of Granzyme B in CD8^+^ T-cells was lower at all time points. **(H)** The percentage of CD8^+^ T-cells expressing Granzyme B was lower at all time points in mice that received Treg.

We next investigated the impact of Treg infusion on the counts and phenotype of Tconv and CD8^+^ T-cells. We observed significantly lower counts of human cells on days 21 (p = 0.008) and 28 (p = 0.02) in mice given non-primed Treg, and on day 21 (p = 0.02) in those given TNF-α primed Treg (**Figure 3A**). Interestingly, there was no benefit of TNF-α priming and, on the opposite, there was a suggestion for higher counts of human cells on day 28 in TNF-α primed Treg compared to unprimed Treg mice (p = 0.05).

Looking at T-cell subtypes/phenotype, we observed that Treg significantly decreased CD8^+^ T-cell counts (**Figure 3B**) and Tconv counts (**Figure 3C**) at days 21 and 28. Furthermore, at day 14, total Tconv in Treg mice had a lower proliferation index (lower KI67) (**Figure 3D**). Regarding Tconv subpopulations, we noticed that the proportion of naive Tconv (defined as CD45RA^+^ CD27^+^ Tconv) among total Tconv was higher in Treg-infused mice at all time points (**Figure 3E**) and that T conv had a reduced activated phenotype (lower HLA-DR) in these mice at day 14 (**Figure 3F**). In addition, Granzyme B expression by CD8^+^ T-cells was significantly lower in Treg-infused than in control mice, again with no impact of TNF-α priming (**Figures 3G, H**).

Taken together, these data demonstrate that Treg infused 48 hours after CD25-depleted hPBMC infusion were successful at decreasing human T-cell proliferation and activation but with no added benefit of TNF-α priming.

### 3.3 TNF-α priming of Treg does not increase their ability at preventing xenogeneic/allogeneic GVHD

**Figure 4:**
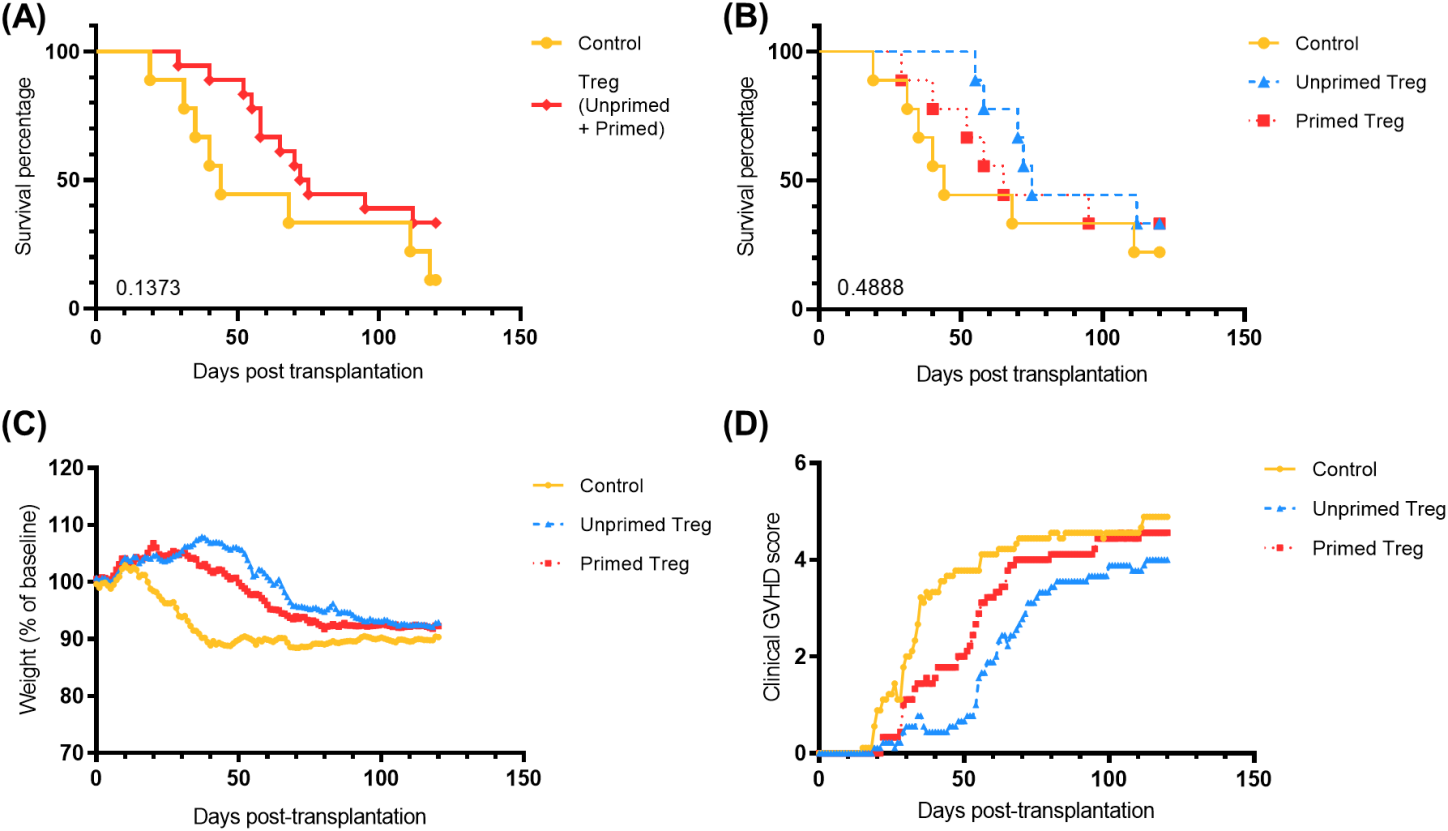
TNF-α priming of human Treg does not increase their ability at preventing GVHD in NSG-HLA-A2/HHD mice injected with human CD25-depleted PBMC. Two million human CD25-depleted PBMC were injected into 27 mice on day 0. Treg were incubated for 48 hours before injecting 500,000 Treg on day 2 (control = no Treg; unprimed Treg = Treg incubated with IL-2 only; primed Treg = Treg incubated with IL-2 and TNF-α at 100 ng/mL). **(A)** Survival curve comparing the control group and the pooled Treg group. **(B)** Survival curve comparing the three groups. Statistical analyses for figures A and B were performed with log-rank (Mantel-Cox) test and did not show significance. **(C)** Weight loss follow-up. **(D)** Clinical GVHD score follow-up. Statistical analyses for figures C and D were performed using two-way Anova and did not show statistical significance.

We next assessed the impact of Treg infusion on x/aGVHD. Comparing survival between control mice and mice injected with Treg, we observed a trend towards longer survival in Treg-injected compared to control mice (HR = 1.9, 95%CI 0.7-5.3, p = 0.14), despite the suboptimal Treg/Tconv ratio (1/4) used (**Figure 4A**). This is in line with prior observations by our group (9) and by other investigators (34). We elected to investigate this suboptimal Treg/Tconv ratio on purpose to be able to assess a potential benefit of TNF-α priming. However, in contrast to what has been observed in mouse-to-mouse models of GVHD (23), there was no apparent benefit of TNF-α priming of Treg in our model since mice receiving TNF-α primed Treg had survival comparable to that of mice receiving unprimed Treg (**Figure 4B**). Similarly, follow-up of weight loss and GVHD scores also evidenced a trend towards a protective effect of Treg but without any significant difference between unprimed and primed Treg (**Figures 4C, D**).

## 4 Discussion

Since *ex vivo* manipulation of Treg can significantly change their suppressive potency and durability in the host, it is essential to have robust and well-characterized *in vivo* models to assess these attributes. The prevailing method to date involves co-transplantation of *in vitro* expanded human Treg together with hPBMCs into NSG mice (10, 34, 35). However, this approach suffers a key limitation: it is inadequate for distinguishing between the infused, experimentally manipulated Treg and the endogenous Treg contained within the transferred PBMC fraction, making it impossible to precisely quantify the persistence, survival, or expansion *in vivo* of only the manipulated Treg population. To circumvent this limitation, we developed a new model allowing the assessment of Treg suppressive potency and persistence *in vivo*. Several observations were made.

First, following infusion of CD25-depleted PBMC, very few Treg are present in the peripheral blood of mice 14–28 days after infusion. This demonstrates that Tconv do not convert into Treg in that model and that the few Treg infused with the CD25-depleted product do not significantly expand.

The most important observation is that Treg infused at a 1 Treg/4 PBMC ratio significantly engraft in NSG mice and persist more than 26 days after infusion. As a consequence, the vast majority of Treg in the blood of mice infused with both CD25-depleted PBMC and Treg originate from the “experimental” Treg infused. This is of importance because it allows to study their phenotype (and/or their transcriptome) at several time points after Treg infusion, and to determine how “experimental” Treg behave *in vivo* following *ex vivo* manipulation.

A third observation was that Treg infused two days after CD25-depleted hPBMC transplantation decreased human Tconv and CD8^+^ T-cell engraftment and/or expansion. Furthermore, Tconv and CD8^+^ T-cells in Treg mice also harbored a less activated phenotype. Finally, despite the suboptimal Treg/Tconv ratio selected by purpose in our study (to assess whether TNF-α priming would increase their efficacy), Treg infusion was associated with a trend towards longer survival. This is in concordance with several studies performed in mouse-to-mouse models of GVHD (6–8), in humanized mouse models of GVHD (9, 11, 12), and in patients given T-cell-depleted HLA-haploidentical transplantation plus additional Tconv (12), HLA-matched T-cell-depleted HLA-haploidentical transplantation plus additional Tconv (14), or double umbilical cord blood transplantation (18).

Using a mouse-to-mouse model of GVHD, Leclerc et al. were the first to demonstrate that GVHD prevention by donor Treg depended on their activation by TNF-α (22). Accordingly, Pierini et al. reported that TNF-α priming of donor Treg increased their ability to prevent experimental GVHD in another mouse-to-mouse model of GVHD (23). These observations prompted us to assess whether TNF-α priming of human Treg would increase their ability to prevent GVHD in the HuCD25^neg^-PBMC-NSG-Treg model.

*In vitro*, we observed a significant impact of TNF-α priming on Treg. Indeed, spectral flow cytometry experiments demonstrated higher expression of TIM3, GARP, CD25, HLA-DR and CD39 in TNF-α primed Treg. The overexpression of GARP seems particularly relevant since the administration of anti-GARP antibodies has been shown to inhibit the suppressive function of Treg in NSG mice infused with human PBMC (11).

Furthermore, single cell RNA sequencing demonstrated substantial gene modelling in response to TNF-α. Indeed, 11 Hallmark pathways including interferon-gamma response, TNF-α signaling via NF-kB, interferon-alpha response, IL6-JAK-STAT3 signaling, inflammatory response, allograft rejection, oxidative phosphorylation, UV response UP, MTORC1 signaling, apoptosis and IL2-STAT5 signaling were significantly upregulated following TNF-α priming. These results are in line with those reported by di Ricco et al. (36) who observed that TNFR2 stimulation induced several signaling pathways such as TNF-α signaling pathway via NF-kB, T-cell activation, cytokine signaling in the immune system and cytokine production. We observed that seven of these 11 pathways were upregulated both in naive and memory Treg subsets. In contrast, oxidative phosphorylation and MTORC1 signaling pathways were only upregulated among naive Treg.

Interestingly, *in vivo*, we observed that TNF-α priming of Treg did not provide any benefit in that model. These results contrast with prior observations by Pierini et al. (23). Possible reasons for these discrepancies might include divergence between mouse and human Treg biology (1), or different study design (Treg infused on day 0 in the study by Pierini et al. versus on day 2 in our humanized study). In addition, while Pierini et al. infused pure Treg (selected by their expression of FoxP3), we infused a population of CD4^+^ T-cells enriched for Treg with a Treg purity (assessed by their FoxP3 expression) ranging from 75 to 90 percent.

As stated above, the disparity between the significant impact of TNF-α priming *in vitro*, observed in spectral flow cytometry and single cell RNA sequencing, and the absence of enhanced function *in vivo*, highlights the absolute need for reliable *in vivo* models to validate *ex vivo* manipulation techniques prior to clinical trials.

Some limitations of our model may be stated. As described above, our product did not contain 100 percent of Treg and the use of the EasySep™ Human CD4^+^CD127^low^CD25^+^ Isolation kit (Stemcell Technologies) provided Treg for 2 mice only per blood donor. Therefore, a different selection technique could help achieve a higher purity and a higher count of Treg. That would allow to transplant more mice per blood donor. In addition, we infused Treg at day 2 following the method that Pierini et al. validated for TNF-α priming of Treg (23) but we did not evaluate other infusion time points which could be relevant for longer *ex vivo* Treg manipulation techniques. Finally, the lack of tissue-level analysis is a limitation of our manuscript although a recent paper by the team of José L. Cohen nicely demonstrated a strong correlation between the clinical grading of xGVHD and the histological tissue grading of GVHD (37).

In summary, we report here the development of the HuCD25^neg^-NSG-Treg model which allows to assess Treg suppressive potency, phenotype and persistence *in vivo.* Using that model, we observed that TNF-α priming of human Treg did not increase their suppressive potency *in vivo*.

## Data Availability

The data presented in the study are deposited in the Biostudies repository (https://www.ebi.ac.uk/biostudies/), accession number E-MTAB-15881.
