# Supplementary material for: Development of a humanized mouse model of graft-versus-host disease to assess human regulatory T cell function
Source: Front Immunol. 2025 Dec 11;16:1717133. doi: 10.3389/fimmu.2025.1717133 (PMC12738934; doi:10.3389/fimmu.2025.1717133)
Supplement: Supplementary file 1 [file DataSheet1.docx]

Supplementary Tables

| **PBMC isolation** | | | | | |
| --- | --- | --- | --- | --- | --- |
| **Reagent** | **Volume** | | | **Company** | **Catalog number** |
| Ficoll-Paque™ Plus | 15 mL in 50 mL Falcon tube | | | Cytiva | 17144003 |
| Dulbecco’s Phosphate Buffered Saline (PBS) | 15 mL in 50 mL Falcon tube for density centrifugation, then 50 mL per wash (3X) | | | Biowest | L0615 |
| **CD25+ cells and Treg isolation** | | | | | |
| **Reagent** | **Volume** | | | **Company** | **Catalog number** |
| EasySep™ Human CD4+CD127lowCD25+ Regulatory T Cell Isolation Kit |  | | | Stemcell Technologies | 18063 |
| EasyEight™ EasySep™ Magnet |  | | | Stemcell Technologies | 18103 |
| Dulbecco’s Phosphate Buffered Saline (PBS) | 98% for recommended medium required for the kit | | | Biowest | L0615 |
| Fetal Bovine Serum (FBS) | 2% in PBS for recommended medium required for the kit | | | Gibco™,  Thermo Fischer Scientific | 10270106 |
| **Culture medium** | | | | | |
| **Reagent** | **Final Concentration** | | **Volume** | **Company** | **Catalog Number** |
| RPMI 1640 Medium | 93 % | | 46.5 mL | Gibco™,  Thermo Fischer Scientific | 22400-089 |
| Human Serum | 5 % | | 2.5 mL | Sigma-Aldrich | H4522 |
| L-Glutamine 200 mM | 1% (2mM) | | 500 µL | Gibco™,  Thermo Fischer Scientific | 25030081 |
| Penicillin 5000 U/mL, Streptomycin 5000 µg/mL | 1% | | 500 µL | Gibco™,  Thermo Fischer Scientific | 15070063 |
| **Cytokines** | | | | | |
| **Reagent** | **Stock concentration** | **Final concentration** | | **Company** | **Catalog Number** |
| Recombinant Human IL-2 | 200 µg/mL | 10 ng/mL | | Peprotech ®,  Thermo Fischer Scientific | 200-02-50UG |
| Recombinant Human TNF-α | 200,000 ng/mL | 10 ng/mL or 100 ng/mL | | Peprotech ®,  Thermo Fischer Scientific | 300-01A-50UG |
| **T cell activation** | | | | | |
| **Reagent** | **Volume** | | | **Company** | **Catalog Number** |
| Dynabeads™ Human T-Activator CD3/CD28 for T-Cell Expansion and Activation | 2 µl in 200 µL of medium per well of 80,000 T cells | | | Gibco™,  Thermo Fischer Scientific | 11131D |

**Supplementary Table 1.** List of materials used in cell culture for PBMC isolation and Treg selection and culture.

| **Antibody** | **Fluorochrome** | **Clone** | **Volume** | **Company** | **Catalog number** |
| --- | --- | --- | --- | --- | --- |
| Fc blocker |  |  | 2 µL in 48 µL  of staining | Biolegend | 422302 |
| **First extracellular staining** | | | | | |
| CD39 | PeCy7 | A1 | 1 µL | Biolegend | 328211 |
| CD223 (LAG-3) | PerCP efluor 710 | 3DS223H | 5 µL | Thermo Fischer Scientific | 46-2239-42 |
| CD366 (TIM3) | BV750 | F38-2E2 | 5 µL | Biolegend | 345056 |
| LAP (TGF-β1) | FITC | S20006A | 2 µL | Biolegend | 300010 |
| CD26 | PE-CF594 | M-A261 | 1 µL | BD Biosciences | 565158 |
| GARP (LRRC32) | APC | 7B11 | 2 µL | Biolegend | 352506 |
| CD54 (ICAM-1) | BUV496 | HA58 | 1 µL | BD Biosciences | 741152 |
| Brilliant Stain Buffer |  |  | 33 µL  (total of 50 µL) | BD Biosciences | 563794 |
| **Second extracellular staining** | | | | | |
| CD4 | Spark Blue 550 | SK3 | 2 µL | Biolegend | 344656 |
| CD25 | BUV395 | 2A3 | 1 µL | BD Biosciences | 564034 |
| CD127 | APC R 700 | HIL-7R-M21 | 4 µL | BD Biosciences | 565185 |
| CD45RA | BV785 | HI100 | 1 µL | Biolegend | 304140 |
| CD45RO | PE/Fire 640 | UCHL1 | 5 µL | Biolegend | 304264 |
| HLA-DR | BV570 | L243 | 2 µL | Biolegend | 307638 |
| CD69 | PerCP | FN50 | 2.5 µL | Biolegend | 310928 |
| CD279 (PD1) | BV650 | EH12.2H7 | 2 µL | Biolegend | 329950 |
| CD278 (ICOS) | BV711 | DX29 | 2 µL | BD Biosciences | 563833 |
| CD120b (TNFR2) | PE | hTNFR-M1 | 5 µL | BD Biosciences | 552418 |
| TIGIT | BV421 | 741182 | 1 µL | BD Biosciences | 747844 |
| Brilliant Stain Buffer |  |  | 22.5 µL  (total of 50 µL) | BD Biosciences | 563794 |
| **Fixable Viability Dye** | | | | | |
| Fixable Viability Dye (FVD) | Efluor 780 | 0.2 µL in 200 µL of PBS | | Thermo Fischer Scientific | 65-0865-14 |
| **Fixation/Permeabilization** | | | | | |
| Foxp3/Transcription Factor Staining Buffer Set | | | | Invitrogen™,  Thermo Fischer Scientific | 00-5523-00 |
| **Intracellular staining** | | | | | |
| CD152 (CTLA-4) | BV605 | BNI3 | 0.5 µL | Biolegend | 369610 |
| FOXP3 | Spark NIR 685 | 206D | 5 µL | Biolegend | 320130 |
| HELIOS | Pe-Cyanine5 | 22F6 | 2 µL | Thermo Fischer Scientific | 15-9883-42 |
| Washing solution |  |  | 42.5 µL  (total of 50 µL) |  |  |

**Supplementary Table 2.** List of antibodies used for the 22-color panel used in spectral flow cytometry for the analysis of *in vitro* experiments.

| **Reagent** | **Final concentration** | **Company** | **Catalog number** |
| --- | --- | --- | --- |
| 3’ CellPlex Kit Set A, 48 rxns |  | 10x Genomics | 1000261 |
| Dulbecco’s Phosphate Buffered Saline (PBS) |  | Biowest | L0615 |
| Bovine Serum Albumine (BSA) | 0.04% or 1% according  to kit protocol | Sigma-Aldrich | A8806 |
| Chromium Next GEM Single Cell 3’Reagent Kits v3.1 |  | 10x Genomics | CG000390 |
| Chromium Next GEM Chip G Single Cell Kit |  | 10x Genomics | 1000120 |
| DynaBeads MyOne SILANE |  | 10x Genomics | 2000048 |
| SPRIselect  Bead-Based Reagent |  | Beckman Coulter | B23317 |
| Dual Index Kit TT, Set A |  | 10x Genomics | PN-3000431 |
| Dual Index Kit NN, Set A |  | 10x Genomics | PN-3000482 |
| KAPA Library Quantification Kit |  | Roche | 07960140001 |

**Supplementary Table 3.** List of materials used for cell multiplexing and library preparation for single cell RNA sequencing.

| **Antibody** | **Fluorochrome** | **Clone** | **Volume** | **Company** | **Catalog number** |
| --- | --- | --- | --- | --- | --- |
| **Extracellular staining** | | | | | |
| CD8 | FITC | HIT8a | 1 µL | eBioscience™,  Thermo Fischer Scientific | 11-0089-42 |
| CD127 | AlexaFluor700 | HIL-7R-M21 | 2 µL | BD Biosciences | 565185 |
| CD4 | BV786 | SK3 | 1 µL | BD Biosciences | 563877 |
| CD25 | BUV395 | 2A3 | 1 µL | BD Biosciences | 564034 |
| Brilliant Stain Buffer |  |  | 45 µL  (total of 50 µL) | BD Biosciences | 563794 |
| **Fixation/Permeabilization** | | | | | |
| Foxp3/Transcription Factor Staining Buffer Set | | |  | Invitrogen™, Thermo Fischer Scientific | 00-5523-00 |
| **Intracellular staining** | | | | | |
| FOXP3 | PECF594 | 259D/C7 | 3 µL | BD Biosciences | 562421 |
| Washing solution |  |  | 47 µL  (total of 50 µL) |  |  |

**Supplementary Table 4.** 5-color panel used in conventional flow cytometry to assess the purity of Treg.

| **Red Blood Cell Lysis** | | | | | |
| --- | --- | --- | --- | --- | --- |
| **Reagent** | | **Dilution** | **Volume** | **Company** | **Catalog number** |
| Red Blood Cell Lysis Buffer  (Multi-species) | | 10x in deionized water | 2 mL of the dilution on  150 µL of blood per sample | eBioscience™,  Thermo Fischer Scientific | 00-4300-54 |
| **Flow Cytometry Panel** | | | | | |
| **Antibody** | **Fluorochrome** | **Clone** | **Volume** | **Company** | **Catalog number** |
| **Extracellular staining** | | | | | |
| CD45h | BV510 | H130 | 1 µL | BD Biosciences | 563204 |
| CD45s | PECy5 | 30-F11 | 0,5 µL | Thermo Fischer Scientific | 15-0451-81 |
| CD4 | APC | RPA-T4 | 0,5 µL | Thermo Fischer Scientific | 17-0049-42 |
| CD8 | PE-Cy7 | SK1 | 1 µL | Biolegend | 344711 |
| CD45RA | BV785 | HI100 | 1 µL | Biolegend | 304140 |
| CD27 | BV650 | L128 | 1 µL | BD Biosciences | 563228 |
| CD62L | BB515 (=FITC) | SK11 | 2 µL | BD Biosciences | 565037 |
| CD25 | BUV395 | 2A3 | 1 µL | BD Biosciences | 564034 |
| HLA-DR | BV605 | L243 | 2 µL | Biolegend | 307640 |
| Brilliant Stain Buffer |  |  | 40 µL  (total of 50 µL) | BD Biosciences | 563794 |
| **Fixable Viability Dye** | | | | | |
| Fixable Viability Dye (FVD) | Efluor 780 |  | 0.2 µl in 200 µl of PBS | Thermo Fischer Scientific | 65-0865-14 |
| **Fixation/Permeabilization** | | | | | |
| Foxp3/Transcription Factor Staining Buffer Set | | | | Invitrogen™,  Thermo Fischer Scientific | 00-5523-00 |
| **Intracellular staining** | | | | | |
| Granzyme B | PE | GB11 | 1 µL | BD Biosciences | 561142 |
| FOXP3 | PECF594 | 259D/C7 | 3 µL | BD Biosciences | 562421 |
| KI67 | Alexafluor 700 | B56 | 1 µL | BD Biosciences | 561277 |
| Washing solution |  |  | 45 µL  (total of 50µl) |  |  |

**Supplementary Table 5.** List of materials and antibodies used for the 13-color panel in conventional flow cytometry for the analysis of mouse blood samples.

Supplementary Figures


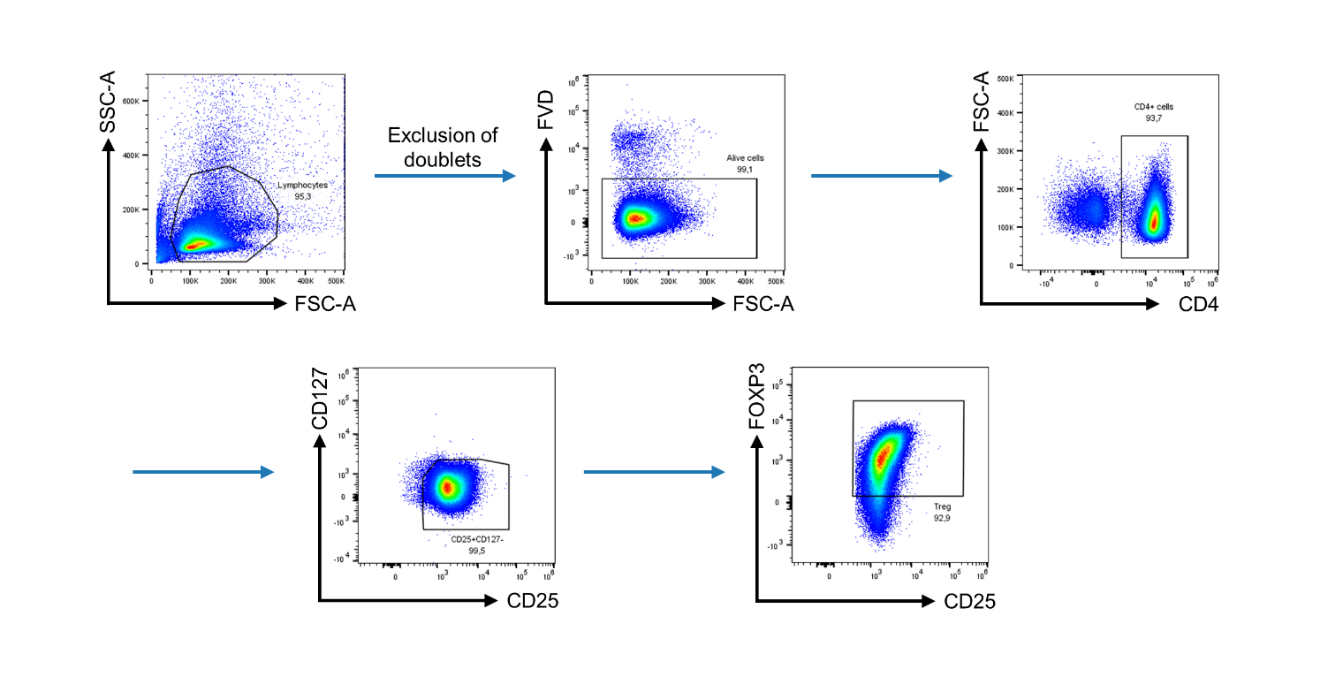


**Supplementary figure 1. Isolation of Treg.**

PBMC were first isolated from buffy coats by Ficoll™ Paque Plus density centrifugation. Treg were selected through immuno-magnetic selection with the EasySep™ Human CD4^+^CD127^low^CD25^+^ Isolation kit. Treg purity ranged from 75 to 90%. In the example shown, there were 86.6% of Treg among alive cells (93.7% CD4^+^ cells x 99.5% CD25^+^CD127^-^ cells x 92.9% FOXP3^+^ cells).


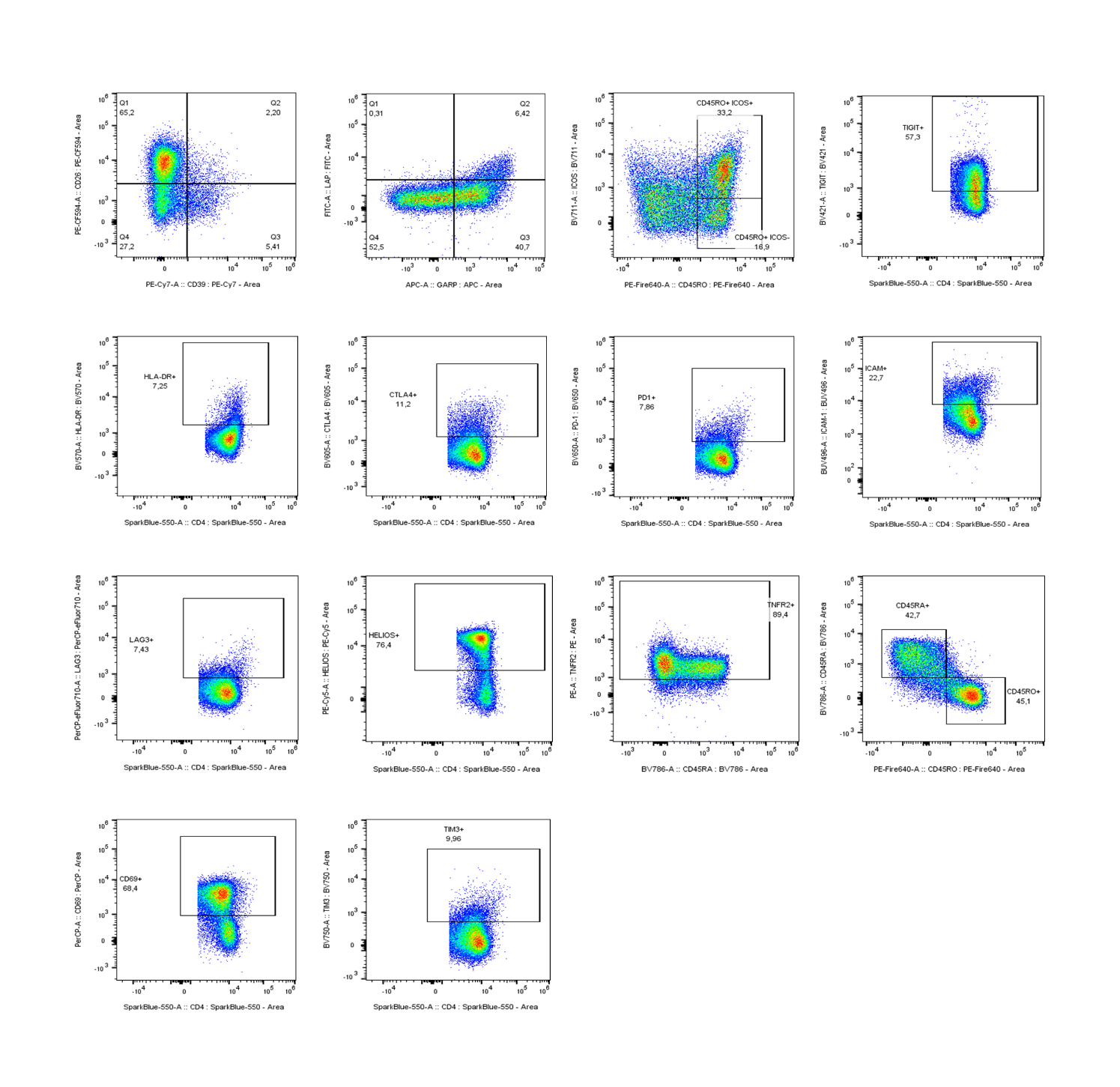


**Supplementary figure 2. Gating strategy for supervised analysis in spectral flow cytometry.**

The gating on Treg was shown on Supplementary Figure 1. The following gating strategy was used after gating on Treg. Of note, fluorescence minus one (FMO) controls were used to ensure accurate gate placement (data not shown).


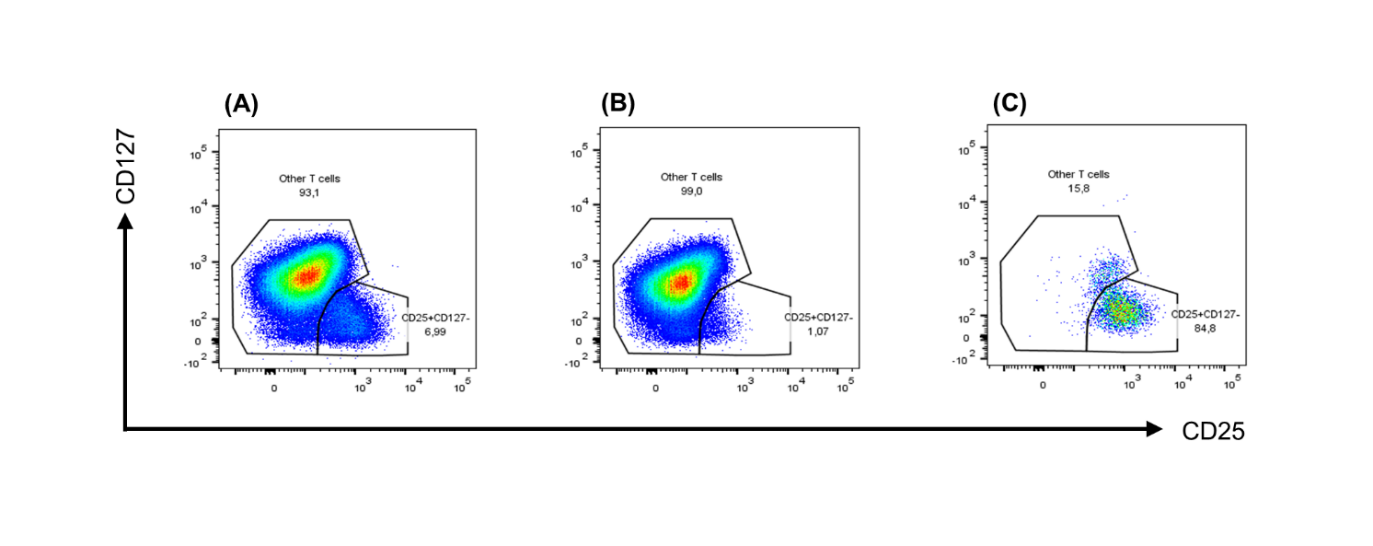


**Supplementary figure 3. The protocol from the EasySep™ Human CD4**^+^**CD127**^low^**CD25**^+^ **Isolation kit was modified in order to deplete CD25**^+^ **cells from PBMC while still isolating Treg.**

**(A)** Total PBMC prior to depletion **(B)** CD25-depleted PBMC **(C)** Isolated Treg


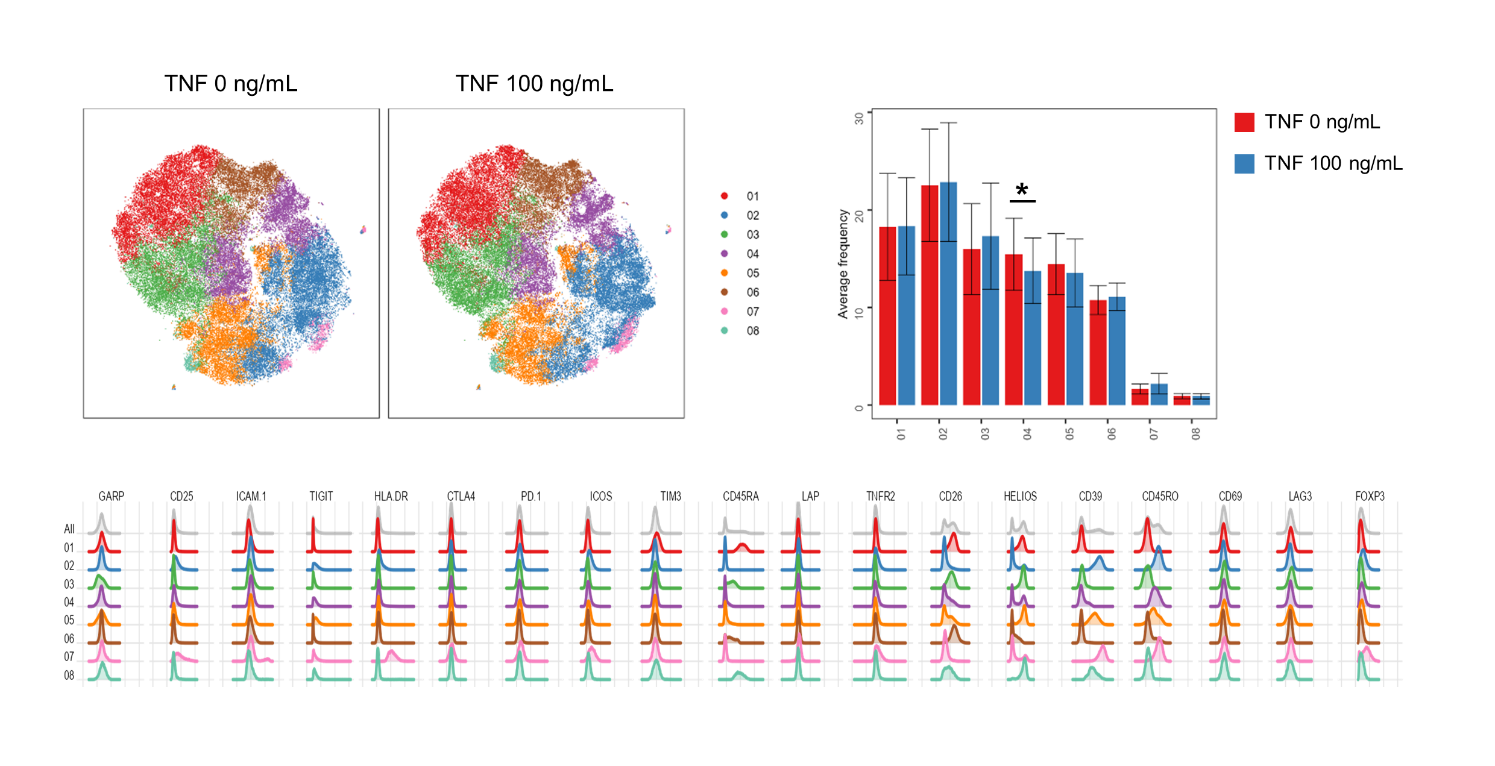


**Supplementary figure 4. Unsupervised analysis on data from TNF-α priming of human Treg not stimulated with CD3/CD28 beads (Spectral flow cytometry).**

In the unsupervised analysis, 8 clusters were defined based on the expression of markers: naive Treg (clusters 1, 3, 6 and 8), M1 memory Treg (cluster 4), M3 memory Treg (cluster 5) and M4 memory Treg (clusters 2 and 7). TNF is expressed in ng/mL.

Statistics were made using the Wilcoxon matched-pairs signed rank test (* = p <0.05).

The frequency of cluster #4, defined as M1 memory Treg (CD45RO^+^CD26^+^CD39^-^ Treg), was significantly lower in Treg that had been exposed to TNF-α (p = 0.031). There was no significant difference in the frequency of other clusters.


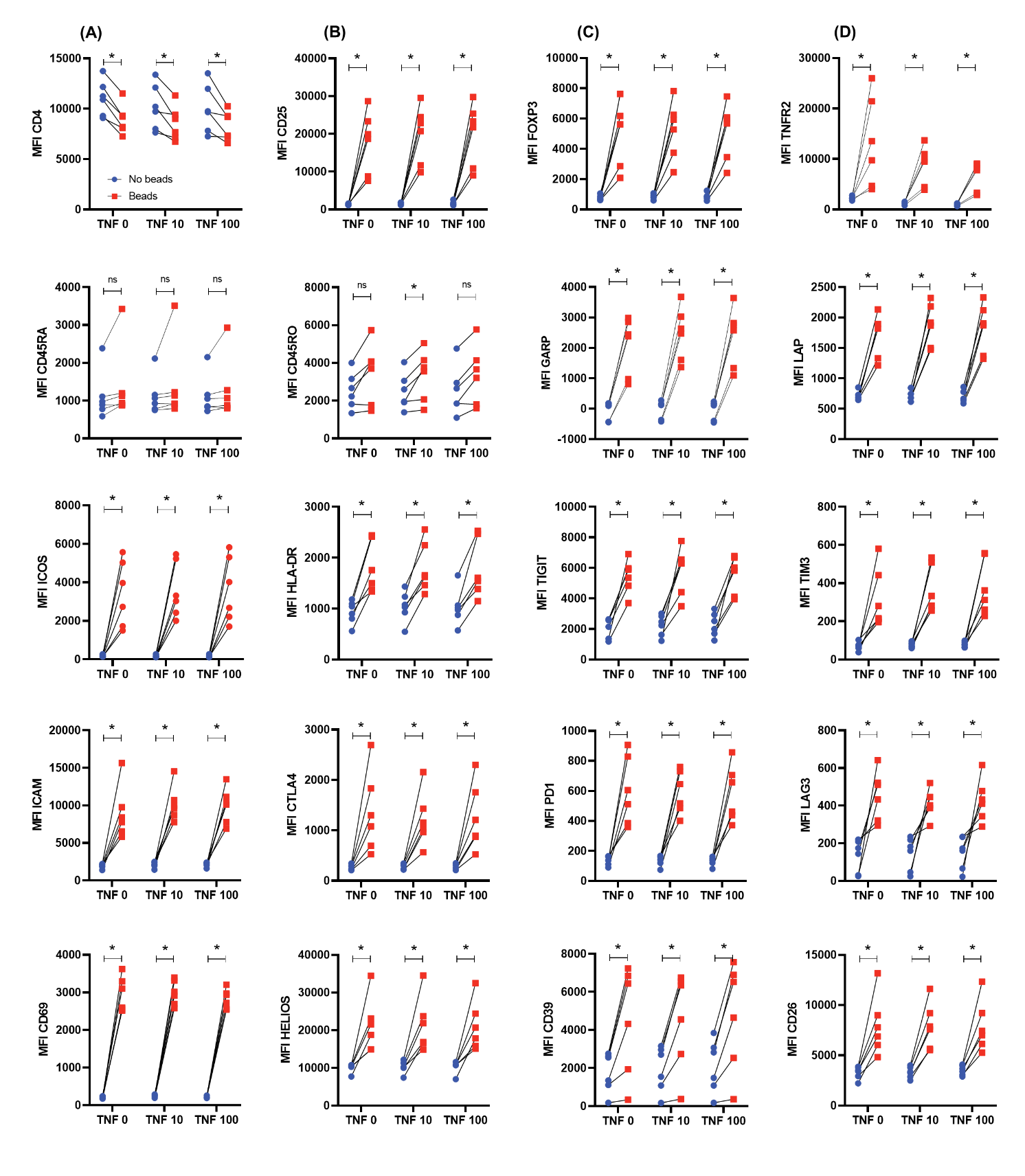


**Supplementary figure 5. Effect of CD3/CD28 beads on Treg phenotype.**

CD3/CD28 beads increased basal expression of most markers and were necessary to observe the effect of TNF-α on some markers such as GARP, CTLA4, PD1, TIM3, LAP, CD69 and LAG3.

**(A)** CD4 is the only marker with decreased expression upon stimulation with CD3/CD28 beads.

**(B)** Effect on the expression of CD25 in CD4^+^ T-cells.

**(C)** Effect on the expression of FOXP3 in CD25^+^CD127^-^ cells.

**(D)** Effect on other markers in Treg (defined as CD4^+^CD25^+^CD127^low^FOXP3^+^ cells).


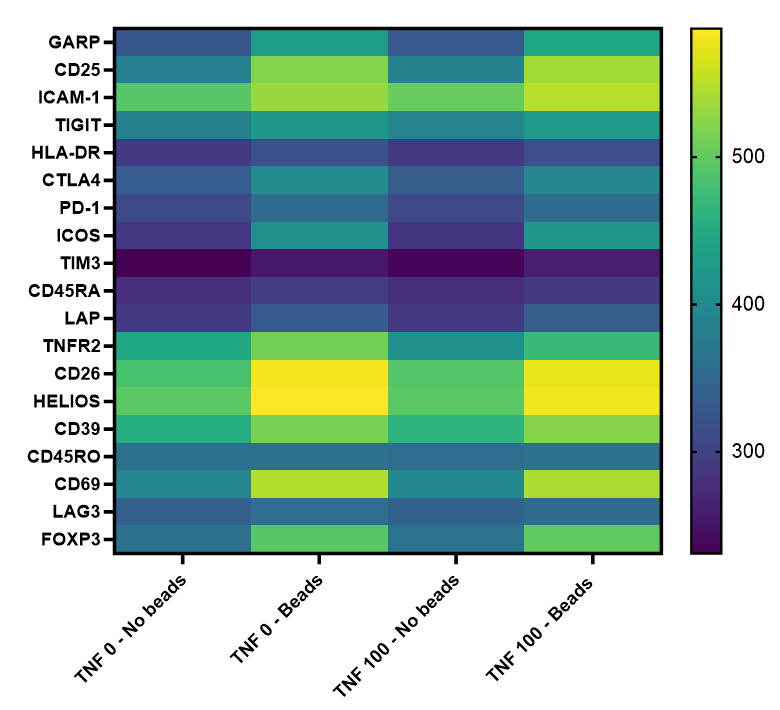


**Supplementary Figure 6. Unsupervised analysis on the effect of CD3/CD28 beads on Treg phenotype.**

Data shown in the heatmap represents the mean fluorescence intensity (MFI) for each marker. Statistical analyses were performed with a Wilcoxon matched-pairs signed rank test.

In Treg which have not been primed with TNF-α (TNF 0), the impact of beads was significant (p < 0.05) on most markers except TNFR2 (p = 0.052), HLA-DR (p = 0.055), CD39 (p = 0.27), CD45RA (p = 0.75) and CD45RO (p = 0.88). In Treg that have been primed with TNF-α (TNF 100), the impact of beads was significant (p < 0.05) on most markers expect HLA-DR (p = 0.09), LAG3 (p = 0.10), CD39 (p = 0.33), CD45RA (p = 0.81) and CD45RO (p = 0.86).

**
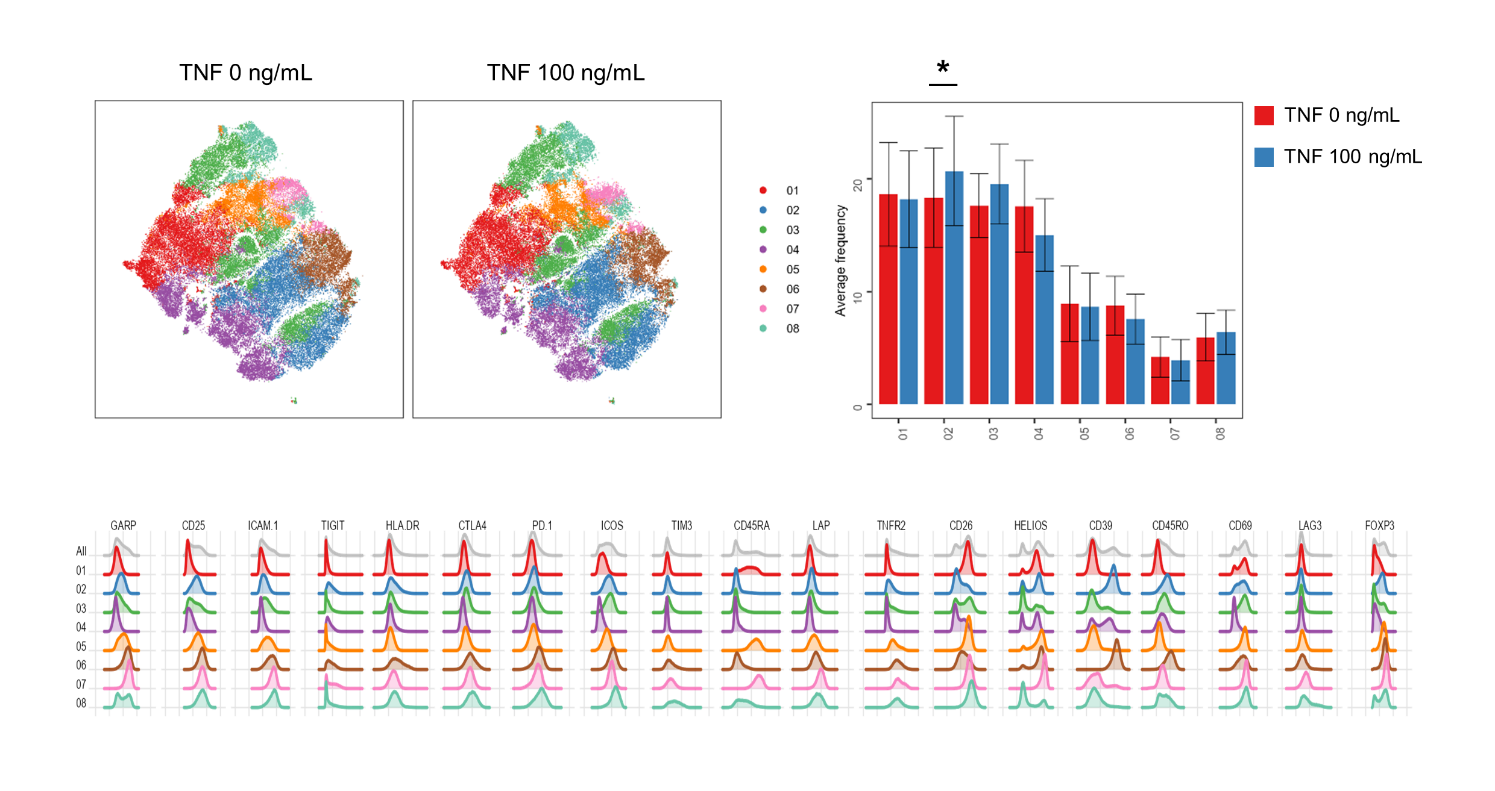
**

**Supplementary figure 7. Unsupervised analysis on data from TNF-α priming of human Treg stimulated with CD3/CD28 beads (Spectral flow cytometry).**

In the unsupervised analysis, 8 clusters were defined based on the expression of markers: naive Treg (clusters 1, 5 and 7), M1 memory Treg (clusters 3 and 8), M3 memory Treg (cluster 6) and M4 memory Treg (clusters 2 and 4). TNF is expressed in ng/mL.

Statistics were made using the Wilcoxon matched-pairs signed rank test (* = p < 0.05).

The frequency of cluster #2 was significantly higher in Treg that had been exposed to TNF-α. There was no significant difference in the frequency of other clusters, although there was a suggestion for a higher proportion of cluster #3 (p = 0.09) and lower proportion of cluster # 4 (p = 0.09).

**
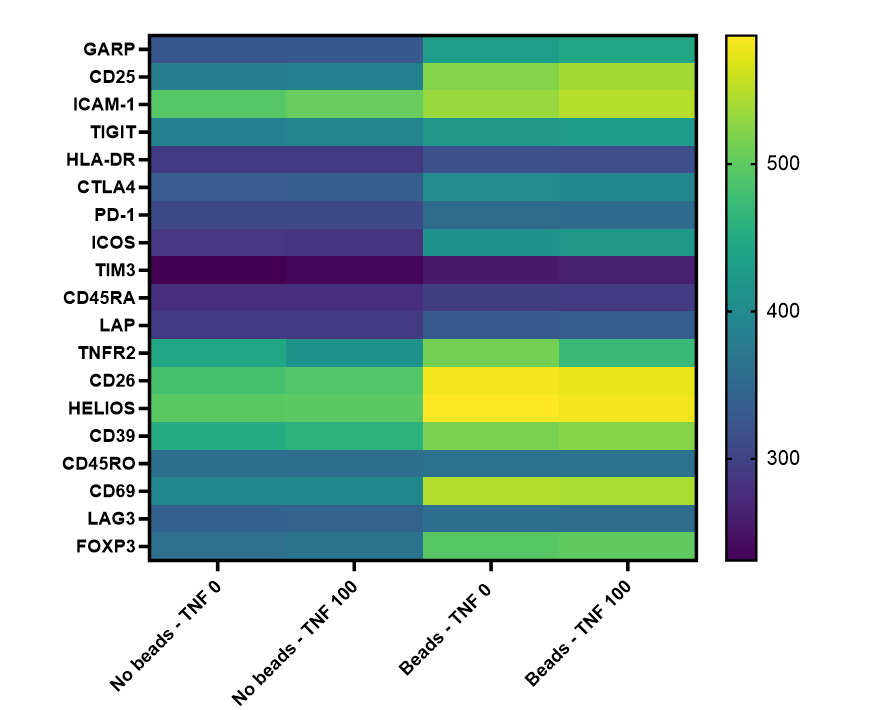
**

**Supplementary Figure 8. Unsupervised analysis on the effect of TNF-α on Treg phenotype.**

Data shown in the heatmap represents the mean fluorescence intensity (MFI) for each marker. Statistical analyses were performed with a Wilcoxon matched-pairs signed rank test.

In Treg not stimulated with beads, the impact of TNF-α was significant on the expression of ICAM (p = 0.036) and TNFR2 (p = 0.031) and close to significance for TIM3 (p = 0.058), TIGIT (p = 0.058), CD69 (p = 0.058), CD26 (p = 0.059) and FOXP3 (p = 0.0625). In Treg stimulated with beads, the impact of TNF-α was significant on the expression of GARP (p = 0.031), CD25 (p = 0.036) and TNFR2 (p = 0.031) and close to significance for TIGIT (p = 0.059) and TIM3 (p = 0.062).

**
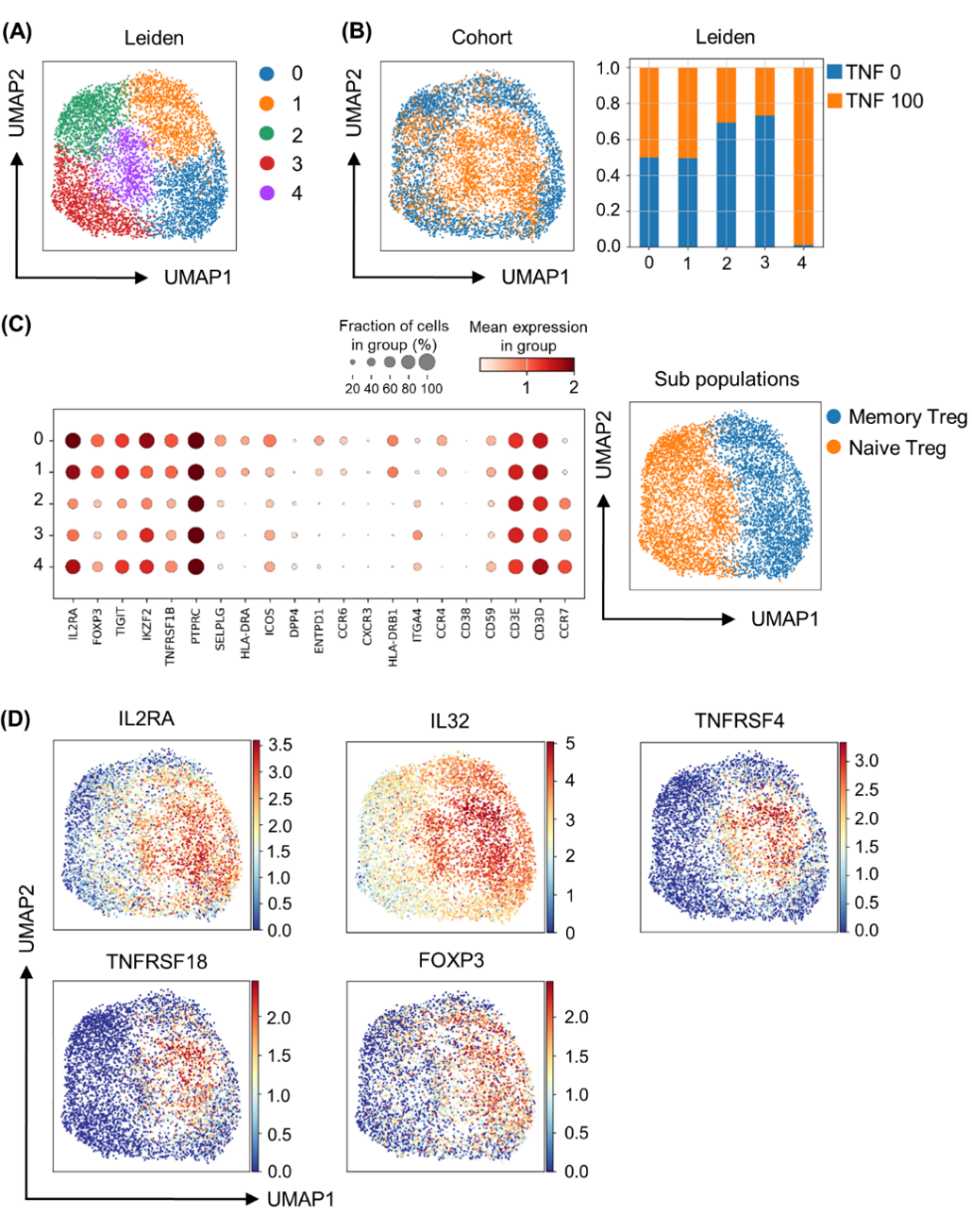
**

**Supplementary Figure 9.** **TNF-α priming induced interesting gene modeling in Treg.**

Treg isolated from 3 PBMC donors were incubated with or without TNF-α at 100 ng/mL for 24 hours. Samples were labelled individually and processed by the Genomic Platform. Data were pooled for analyses in a control group and a TNF-α primed Treg group.

**(A**) UMAP representation of all samples after filtering out Treg. Clustering with Leiden algorithm with a resolution of 0.30, we obtained 5 clusters including 3,043 TNF-α primed Treg and 3,326 control Treg (total of 6,369 cells). **(B)** The histogram shows the proportion of control Treg and TNF-α primed (TNF 100) Treg in each cluster. Cluster 4 contains mainly primed Treg, while clusters 2 and 3 contain mainly control Treg. Primed Treg are gathered at the center of the UMAP. **(C)** Regarding gene expression, clusters 0, 1 and 4 which contain mainly TNF-α primed Treg had a higher expression of Il-2RA (CD25), FOXP3 and TIGIT. Clusters 2 and 3 harbored a more naive phenotype. Naive and memory Treg were labelled based on their expression of CCR7 and DPP4. **(D)** UMAP representation of gene expression. TNF-α primed Treg showed a higher expression of IL2RA (CD25), IL32, TNFRSF18, FOXP3 and TNFRSF4. In DEG analysis, IL2RA was significantly upregulated 2.5 fold (adjusted p value < 0.001). FOXP3 is upregulated 1.38 fold but this did not reach statistical significance (p = 0.08). The upregulation of IL32, TNFRSF18 and TNFRSF4 is stronger and statistically significant (adjusted p value < 0.001).


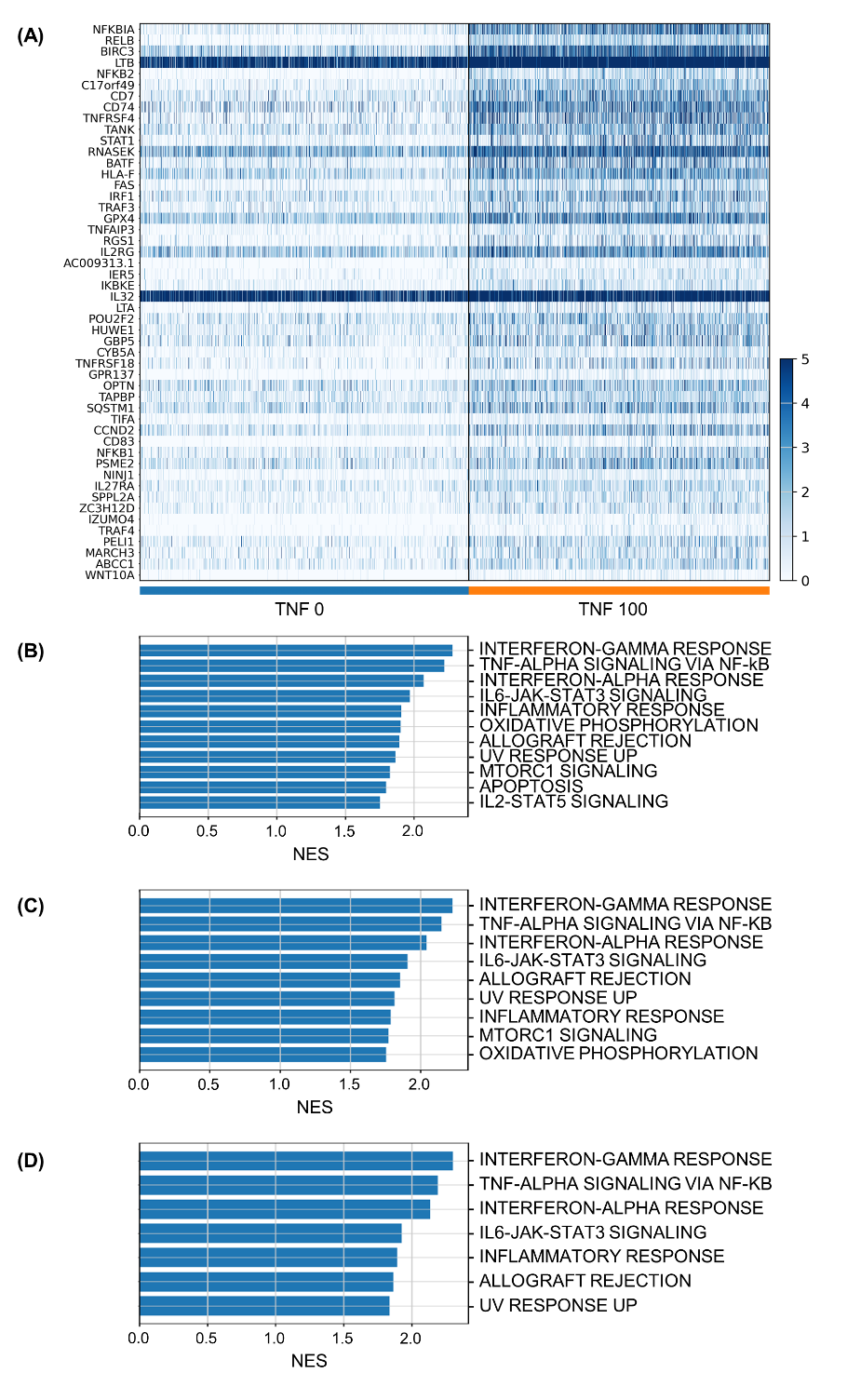


**Supplementary Figure 10. TNF-α modulates gene expression towards Treg activation.**

Treg isolated from 3 PBMC donors were incubated with or without TNF at 100 ng/mL for 24 hours. Samples were labelled individually and processed by the Genomic Platform. Data were pooled for analyses in a control group and a TNF-α primed Treg group.

**(A)** Heatmap of most upregulated genes after exposure to TNF-α. Gene expression levels are log-normalized counts, where 0 indicates low expression and 5 represents high expression. The color representing 5 includes all values greater than or equal to 5. **(B-D)** Genes were ranked by a t-test for GSEA analysis using the Hallmark database. Data shown are significantly upregulated signaling pathways (NES > 1.75 et FDR < 0.01) in Treg in the TNF-α primed group compared to the control group **(B)**, in “naive” treated vs untreated Treg **(C)**, and in “memory” treated vs untreated Treg **(D)**.
